# Diabetic peripheral neuropathy: pathogenetic mechanisms and treatment

**DOI:** 10.3389/fendo.2023.1265372

**Published:** 2024-01-09

**Authors:** Jinxi Zhu, Ziyan Hu, Yifan Luo, Yinuo Liu, Wei Luo, Xiaohong Du, Zhenzhong Luo, Jialing Hu, Shengliang Peng

**Affiliations:** ^1^ Department of Anesthesiology, The Second Affiliated Hospital of Nanchang University, Nanchang, Jiangxi, China; ^2^ The Second Clinical Medical College of Nanchang University, The Second Affiliated Hospital of Nanchang University, Nanchang, Jiangxi, China; ^3^ Department of Sports Medicine, Huashan Hospital, Fudan University, Shanghai, China; ^4^ Department of Emergency Medicine, The Second Affiliated Hospital of Nanchang University, Nanchang, Jiangxi, China

**Keywords:** diabetic peripheral neuropathy, molecular mechanisms, signal transduction, diagnosis, treatment

## Abstract

Diabetic peripheral neuropathy (DPN) refers to the development of peripheral nerve dysfunction in patients with diabetes when other causes are excluded. Diabetic distal symmetric polyneuropathy (DSPN) is the most representative form of DPN. As one of the most common complications of diabetes, its prevalence increases with the duration of diabetes. 10-15% of newly diagnosed T2DM patients have DSPN, and the prevalence can exceed 50% in patients with diabetes for more than 10 years. Bilateral limb pain, numbness, and paresthesia are the most common clinical manifestations in patients with DPN, and in severe cases, foot ulcers can occur, even leading to amputation. The etiology and pathogenesis of diabetic neuropathy are not yet completely clarified, but hyperglycemia, disorders of lipid metabolism, and abnormalities in insulin signaling pathways are currently considered to be the initiating factors for a range of pathophysiological changes in DPN. In the presence of abnormal metabolic factors, the normal structure and function of the entire peripheral nervous system are disrupted, including myelinated and unmyelinated nerve axons, perikaryon, neurovascular, and glial cells. In addition, abnormalities in the insulin signaling pathway will inhibit neural axon repair and promote apoptosis of damaged cells. Here, we will discuss recent advances in the study of DPN mechanisms, including oxidative stress pathways, mechanisms of microvascular damage, mechanisms of damage to insulin receptor signaling pathways, and other potential mechanisms associated with neuroinflammation, mitochondrial dysfunction, and cellular oxidative damage. Identifying the contributions from each pathway to neuropathy and the associations between them may help us to further explore more targeted screening and treatment interventions.

## 1 Introduction

Diabetic neuropathy (DN) is one of the most frequent chronic complications of diabetes mellitus, along with diabetic eye complications, diabetic foot, and diabetic cardiovascular complications. The disease can involve both central and peripheral nerves, particularly the latter, known as DPN, which has been shown to affect about one-third of patients with peripheral neuropathy (1). As the number of diabetic patients increases further worldwide, DPN has become a global health challenge. The aggregate annual cost of treating painful DPN and its complications (such as foot ulcers and limb amputations) in the United States has been estimated to be between $4 billion and $13 billion; up to 27% of direct medical costs for diabetes are attributable to DPN (2).

The commonest manifestation of DPN is distal symmetrical limb numbness with loss of sensation, and about 20% of people with diabetes may also develop neuropathic pain due to DPN. Common types of pain include cauterizing, electrical and sharp pains, followed by pruritus, hyperalgesia, and evoked pain (3). In addition to this, the combination of hyperglycemia and metabolic disorders harms the immune system and immune function of the body, and this unconscious, insidious wound may eventually become infected and lead to serious limb damage (4). Current studies regard DPN as the most common cause of non-traumatic lower limb amputation in most high-income countries (5).

The etiology and pathogenesis of DPN are still inconclusive but are currently thought to be mainly related to a series of pathophysiological processes caused by hyperglycemia, dyslipidemia, and insulin resistance. Abnormal glucose-lipid and insulin resistance and its sequelae cause alterations in mitochondrial function, inflammation, oxidative stress, specific gene transcription, and expression, ultimately leading to neuronal-glial cell damage. In addition, some widely used clinical drugs, such as proton pump inhibitors and metformin, which are commonly used in diabetic patients, may also cause/aggravate DPN by inducing vitamin B12 deficiency (6, 7). This paper reviews the existing research in cellular and animal models to understand the mechanisms of initiation and progression of DPN, which and the associations between them may be useful for early screening, graded treatment, and prognostic assessment of DPN.

## 2 Epidemiology

Considered a major chronic disease and epidemic of our time, diabetes has become the leading cause of death and disease in the global population and poses a continuously growing disease burden for countries around the world. The global prevalence of diabetes is currently increasing year on year, and the rates of screening, treatment, and control are less than optimal. Results of a large diabetes survey based on the mainland Chinese population show that nearly half of the adults have abnormal blood sugar (8). DPN is the most common and most difficult complication of diabetes mellitus (DM) to treat, with the highest morbidity and mortality rates and a huge financial burden on diabetes treatment. Studies have shown that nearly half of all people with diabetes will develop peripheral neuropathy, and the process often begins early in the course of diabetes, with the extent and rate of progression depending on several other factors, including the age of the patient, the number of years they have had diabetes, and the level of blood glucose control (9–12). With recent advances in diagnostic techniques, there is a tendency for this value to increase further when measured by the more sensitive nerve conduction test (13). A multicenter study based on diabetic patients in Beijing showed that the prevalence of DPN in Chinese patients with type 1 diabetes mellitus (T1DM) and type 2 diabetes mellitus (T2DM) was 21.92% and 35.34%, respectively (14).

DSPN is the most common type of DPN. Available studies suggest that DSPN is present in approximately 28% of diabetic patients (15). Another common type of DPN is diabetic autonomic neuropathy, and in a clinical study assessing the prevalence of cardiac autonomic neuropathy (CAN) in a sample of Chinese diabetics, researchers found an overall prevalence of CAN in combination with diabetes of up to 63% (16).

## 3 Mechanism

### 3.1 Overview of the mechanism of DPN

**Figure 1:**
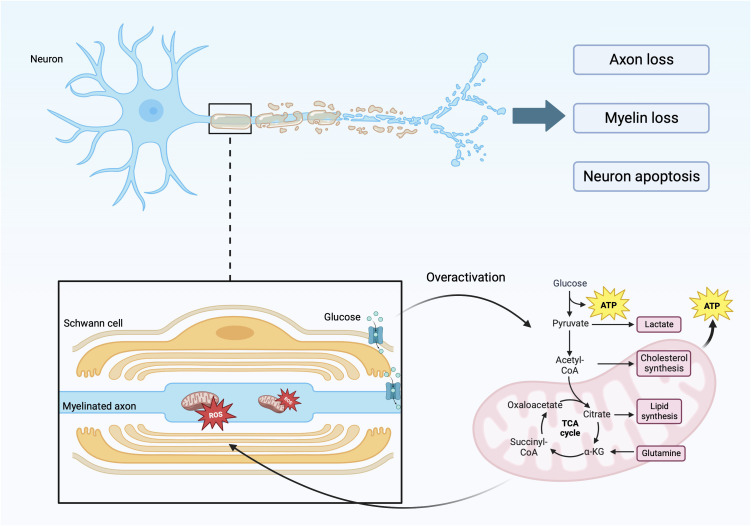
Specific manifestations of axonal myelin sheath injury.

DPN is one of the most common complications of diabetes, which will reduce the patient’s exercise ability. In addition, DPN can also cause painful diseases such as neuropathic pain, diabetic foot and its complications such as foot ulceration, and even increase the risk of lower-limb amputation and death (17–19). DN is characterized by a stocking-glove distribution and distal symmetric polyneuropathy. This is caused by the loss of myelin in myelinated, injury of unmyelinated nerves, axonal atrophy, and other factors, which are manifested as affected nerve conduction velocity and abnormal sensory function (**Figure 1**) (20–22). This disease features result from the specific anatomy of motor-sensory neurons and glial cells in the peripheral nervous system. Peripheral nerves are composed of axons, cytoplasmic processes, and Schwann cells (SCs) (a type of glial cell) (23). Glial cells are important for nerve conduction velocity because they are characteristic of insulating and provide the conditions for rapid, saltatory conduction of action potentials over long distances (24, 25). Myelinated nerve fibers have glial cells wrapped around the periphery of axons, while small axons form non-myelinating Remak bundles (26). The overall organization formed by the myelin sheath and axon has radial polarity and is composed of different inner membranes within it. It is rich in receptors and adhesion molecules that maintain the peri-axonal space and translocate growth factor signals from axons (23, 27, 28). Under the influence of hyperglycemia and hyperlipidemia, and through various signaling pathways, the regulatory functions of SCs, such as cell autophagy and cell metabolism, are damaged and dysfunction of mitochondria (**Figure 1**) (17, 29, 30). Without the protection and support of glial cells, such as SCs, sensory neurons are more vulnerable to injury than motor neurons, especially neurons in the dorsal root ganglion (31, 32). The structure and function of sensory neurons are particularly vulnerable. Because sensory neurons often form unmyelinated nerve fibers, they rarely form myelinated nerve fibers (33). Oxidative stress, metabolic abnormalities, microangiopathy, and other factors caused by diabetes, through special signal transduction pathways, destroy the normal structure and function of nerve cells and lead to neuronal demyelination and neuronal damage, which are the main causes of peripheral neuropathy (34, 35).

Although DPN is a common complication of T1DM and T2DM, many studies have shown that its pathogenesis differs in T1DM and T2DM. Differences in metabolic factors between T1DM and T2DM result in different structural changes in peripheral nerves (36). C-peptide significantly prevents and improves nerve conduction abnormalities in T1DM rats, while no significant alterations were found in type 2 diabetes mellitus (37). In an analysis of transcriptomic data on mouse models of type 1 and type 2 diabetes of the DPN genes, researchers found that genes involved in insulin signaling, endoplasmic reticulum stress, and more are differentially altered in peripheral nerves in T1DM and T2DM. In T1DM mice, the pathogenesis of DPN is more involved in lipid biosynthesis and cholesterol processes, while in T2DM, it is more involved in MAPKinase NF-κB pathways (38). Another study analyzed DPN-related genes and pathways in the sciatic nerve of T1DM and T2DM mice and found that many of the specific differentially expressed genes (DEGs) in T1DM mice are localized in the nucleoplasm and are involved in the regulation of transcriptional processes, whereas the specific DEGs in T2DM mice are located at cellular junctions and are involved in ion transport (39). Different therapeutic effects exist for the same treatment modality for DPN due to the presence of different DPN mechanisms. For example, glycemic control is more effective for T1DM, whereas for T2DM, multifactorial interventions are required (31). In conclusion, although T1DM and T2DM are always discussed together when studying the molecular mechanisms of DPN. However, it should be clear that the study of DPN pathogenesis in different DMs facilitates the discovery and use of more effective treatments.

The figure reflects the specific factors and mechanisms that cause axonal and myelin damage. During the process of axon and myelin sheath damage, there is involvement of glycolysis in mitochondria, leading to excessive production of ROS, leading to functional and metabolic abnormalities in nerve cells.

### 3.2 Oxidative and metabolic pathways

**Figure 2:**
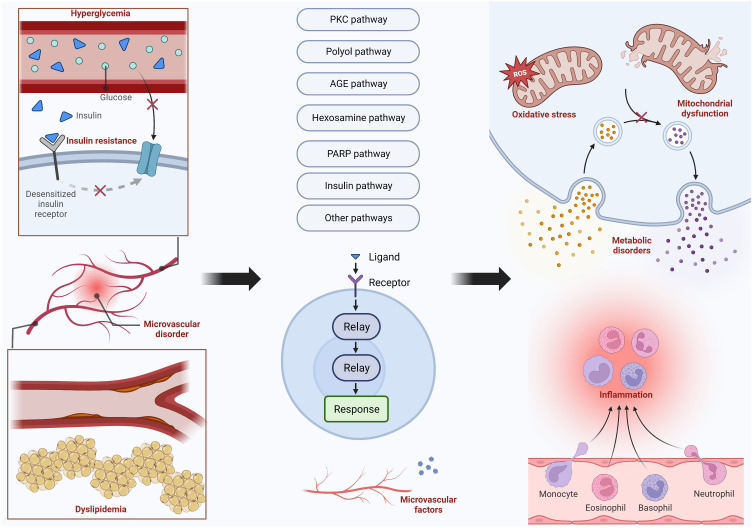
Important Pathways in the Mechanism of DPN.

The damage to nerve cells is often caused by metabolic disorders, oxidative stress, and inflammatory reactions. The mechanisms that cause these impacts include many pathways, and their mechanisms and specific pathways will be presented in the following text. **Figure 2** shows the main pathways and their upstream and downstream influencing factors. In addition, there are many factors that affect the function of the peripheral nervous system, such as central nervous system disorders that may have an impact on the peripheral nervous system.

DPN results from oxidative stress, mitochondrial dysfunction, and other metabolic pathways. Hyperglycemia, dyslipidemia, insulin resistance, and microvascular disorder are the four main factors that lead to DPN. Hyperglycemia and dyslipidemia are the most common two factors that can trigger the PKC pathway, polyol pathway, AGE pathway, hexosamine pathway, and PARP pathway. Insulin pathways, microvascular disorders, and other pathways are also activated to bring some harmful nervous effects, including inflammation, metabolic disorders, oxidative stress, and mitochondrial dysfunction.

#### 3.2.1 Protein kinase C pathway

**Figure 3:**
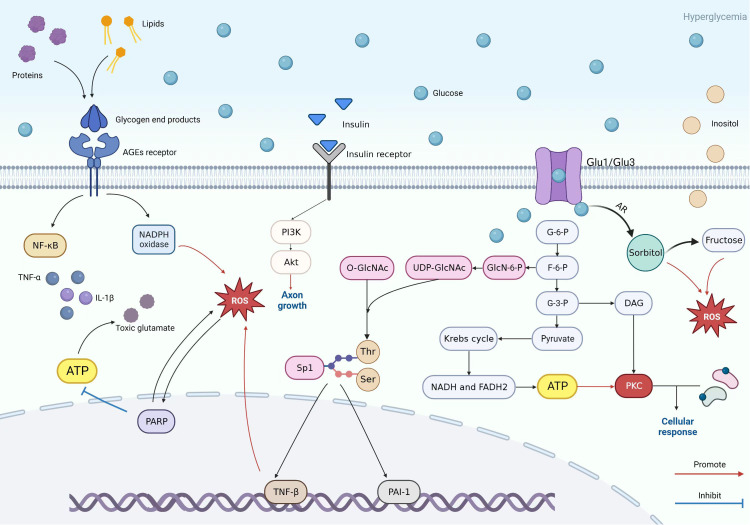
Patterns of PKC pathway, AGE pathway, insulin pathway, polyol pathway, and hexosamine pathway.

Glycolysis is highly involved in glucose metabolism and is a fundamental process in pathways such as the PKC pathway and AGE pathway. Glucose is transported into cells by Glut-1 and Glut-3 to participate in glycolysis. During this process, glucose is gradually phosphorylated and metabolized, resulting in the production of Glucose-6-phosphate, fructose-6-phosphate, glyceraldehyde-3-phosphate, and pyruvate (40). Due to diabetes, the glucose in the blood is abnormally elevated, and the intermediate glyceraldehyde-3-phosphate can be converted into diacylglycerol (DAG), which can activate the neuronal PKC pathway (33). In addition, Glucose-6-phosphate goes through glycolysis to form pyruvate, which enters the mitochondrial Krebs cycle to produce NADH and FADH2 and can then be oxidized to produce ATP. It provides conditions for activating the PKC pathway (**Figure 3**) (41, 42).

PKC is a serine or threonine kinase that binds to Ca^2+^-activated calmodulin and affects the function of other proteins (43). PKC activation causes the activation and phosphorylation of ATPase, causing various metabolic damage and disorders, including altering vascular endothelial growth factors, leading to vasoconstriction, and affecting normal metabolism of the body (44, 45). For example, under abnormal circumstances, β cells’ unique metabolic inventory to regulate insulin production and secretion as normal is broken, and the activation of the PKC pathway can affect the normal metabolism of the body. It’s recently been reported that the PKC pathway affects the normal regulatory effect of guanosine on glial cells, reduces the viability of glial cells and glutamate uptake, and increases the production of reactive oxygen species, leading to oxidative damage (46, 47).

However, the direction of the PKC pathway is not singular, and its impact on neural cells is bidirectional. Recent studies have shown that insulin promotes the growth of axons through PKC-related pathways. In the insulin pathway, insulin serves as a neurotransmitter that can nourish and sensitize sensory nerves (48). Although the mechanism has not been fully explored, it is generally achieved by insulin-activating Ras-related C3 botulinum toxin substrate 1 (Rac1). Rac1 is a small GTP enzyme associated with axonal growth (49). It can be observed that the PKC pathway involves multiple molecular pathways, and how to selectively utilize the PKC pathway is the problem we are facing. PKC inhibitors and activators can be used for PKC-mediated disease treatment. This is also one of the latest targets for the treatment of axonal injury, and treatment methods related to pathogenic signaling pathways are potential therapeutic targets in the future (50).

#### 3.2.2 Polyol pathway

Early neurological dysfunction is reflected in the accumulation of sorbitol in the axons and the dysfunction of Na/K-ATPase (51). The specific mechanism is that excess glucose is converted into sorbitol by aldose reductase (AR). The increase in sorbitol can disrupt the cell osmotic balance. This results in osmotic stress and a compensatory outflow of inositol and taurine. The loss of inositol results in damage to the normal functional structure of nerve cells. Excessive activation of the polyol pathway promotes the occurrence of neuropathy (**Figure 3**) (33). Increased production of reactive oxygen species leads to oxidative stress (52, 53). Sorbitol is then converted to fructose by sorbitol dehydrogenase (54). Studies on diabetes mice and diabetes men show that sorbitol, fructose, and other polyol pathway intermediates promote oxidative damage in animals and patients, leading to neuropathy (55).

Due to the characteristics of SCs, glucose preferentially enters the SCs of the peripheral nerves. It is worth noting that aldose reductase is mainly located in SCs, and high blood sugar will first cause metabolic disorders in SCs, leading to axonal loss of support and protection from SCs and damage (56).

What can be referred to is that recent studies have shown that in the ventricular cells of T2DM rats, the increase of fructose increases the glycolysis ability and cytoplasmic lipid content (57). The mechanism by which excessive fructose causes damage in nerve cells has not yet been explored. Although the polyol pathway is one of the most studied molecular mechanisms, many gaps remain to be explored.

#### 3.2.3 Advanced glycation end pathway

When proteins and lipids are exposed to high blood sugar levels, they form heterogeneous molecules with a high level of reactivity known as glycation end products (58). For example, glycohemoglobin, carboxymethyl arginine, imidazolone, formyl threosyl pyrrole, arg pyrimidine, pentosidine, and crossline (59). AGE pathways are various pathways that use AGEs as triggers. AGEs bind to late glycation end-product receptors to activate chemotactic factors and pro-inflammatory markers, for example, NF-κB, TNF-α, and interleukin, triggering downstream pathways that lead to inflammatory responses (**Figure 3**) (60, 61). Therefore, the accumulation of AGEs leads to a series of inflammatory reactions and causes microvascular damage and glial cell dysfunction. At the same time, it will also activate NADHPH oxidase and increase ROS production. The generated ROS will also promote the process of AGE production (62). The abnormally high levels of ROS in cells over a long period will gradually exacerbate irreversible oxidative stress, leading to cell death (63, 64).

During the process of generating AGEs, intermediate products, namely Amadori compounds, are produced. In the process of forming AGEs from Amadori compounds, 3-deoxyglucosone is key to this pathway (59). The production of 3-deoxyglucosone is closely related to the polyol pathway, and the interaction between these pathways exacerbates oxidative stress.

The AGE pathway has complex mechanisms, which are caused by the complex interactions of various intermediate products in the pathway and the interactions between the AGE pathway and other pathways. It leads to the occurrence of factors that damage nerve cells, such as inflammation and oxidative stress. Currently, more and more evidence suggests that the accumulation of AGEs is independently related to DN, and the therapeutic effect of electroacupuncture on DN also shows a decrease in AGEs and an improvement in neuropathic pain (65, 66). Reducing the generation and accumulation of AGEs is one of the directions for treating DN. In the future, how to suppress the AGE pathway and reduce the generation and accumulation of AGEs is the problem we need to explore.

In addition to what has already been mentioned, fructosylation plays a significant role in advancing the AGE pathway. Through fructose kinase, fructose is transformed into fructose-1-phosphate, which enters glycolysis without going through the major step of glycolysis. Lactic acid, glucose, or fatty acids can be created from the trisaccharide phosphate that fructose-1-phosphate produces. This causes aberrant blood lipid levels and intensifies AGE pathway activation, which promotes neuropathy (67).

#### 3.2.4 Hexosamine pathway

The hexosamine pathway is a molecular pathway induced by hyperglycemia that damages Chevron cells and nerve cells through oxidative stress and inflammation, ultimately causing DPN. In normal conditions, a small part of fructose-6-phosphate from the glycolytic route enters the hexosamine route and is converted to glucosamine-6-phosphate by the action of glutamine fructose-6-phosphate amidotransferase (68). Then, glucosamine-6-phosphate was transformed to uridine diphosphate-n-acetylglucosamine (UDP-GlcNAc). UDP-GlcNAc is an essential primer for O-linked-beta-D-N-acetylglucosamine (O-GlcNAc) transferase, attaching O-GlcNAC to serine and threonine residues of several important transcription factors (e.g., specificity protein 1 (Sp1)) (69). However, in the presence of hyperglycemia, the flux of the hexosamine pathway is increased, which activates the Sp1 pathway. Sp1 can regulate the expression of some glucose-induced “housekeeping” genes, such as fibrinogen activator inhibitor-1 (PAI-1) and transforming growth factor-β (TGF-β) (70, 71). In a controlled experiment, the lack of immunodetection of tissue fibrinogen activator was found to increase the number of peripheral nerve microvascular in the diabetic peroneal outer membrane and intra-neural vessels by four to six times. This result indicates that overexpression of PAI-1 leads to microvascular ischemia as well as thrombosis in diabetic neuropathy (72). TGF-β can induce apoptosis and axonal damage by inducing ROS production (**Figure 3**) (73). Further studies on the expression of several transcription factors and their downstream molecules in the hexosamine pathway will contribute to the discovery of more therapeutic approaches for DPN in the future.

#### 3.2.5 PARP pathway

Poly(ADP-ribose) polymerase (PARP) is a nuclear DNA repair enzyme with multiple regulatory functions (74–76). It is a prominent marker of DPN (77). PARP-1 is the major PARP subtype and is abundant in the nucleus. PARP-1 plays an important role in DNA repair and maintaining the integrity of the genome (75, 78). It also regulates the expression of proteins such as inflammatory mediators, apoptosis, and cell necrosis at the transcriptional level (79, 80). An investigation in Russian patients with T1DM showed a close relationship between PARP-1gene and the pathogenesis of DPN (81). A completely normal intraepidermal nerve fiber density is observed in a diabetic PARP-deficient mouse model (82). In the Akita mouse model, reduced diabetes-related axonal atrophy was observed in mice following the use of GPI-15427, an inhibitor of PARP (83). In both models above, reduced motor nerve conduction velocity and sensory nerve conduction velocity deficits were observed, as well as unaltered hyperglycemia.

PARP has a role in the pathogenesis of DPN through two mechanisms (22). The first mechanism is PARP activation, which affects the rate of ATP production by consuming NAD^+^, leading to peripheral nerve energy deficiency, as well as the accumulation of toxic glutamate causing slowed nerve conduction and degeneration of myelinated nerve fibers (84). The second mechanism consists mainly of poly(ADP-ribosyl)ation affecting transcriptional regulation and gene expression, which is associated with multiple hyperglycemia-related pathways as well as oxidative stress and nitrosative stress (**Figure 3**) (80, 85).

Hyperglycemia inhibits glyceraldehyde-3-phosphate dehydrogenase (GAPDH) activity and slows down glycolysis as a result of poly(ADP-ribosyl)ation of GAPDH by PARP. This ultimately causes the activation of the PKC pathway, the increased flux of the hexosaminidase pathway, and the production of the AGE pathway (86). The mechanisms by which the three aforementioned pathways play a role in DPN are mentioned in the earlier part of the article. The oxidative stress and nitrosative stress-PARP pathways also play a key role in the development of DPN. It is now suggested that PARP activation is triggered not only by free radical and oxidant production but also leads to free radical and oxidant production. This suggests that oxidative/nitrosative stress and PARP activation interact in diabetes (87). On the one hand, hyperglycemia-induced oxidative stress-mediated induction of DNA single-strand breaks is thought to be a signature of PARP activation. Some experiments observed diabetes-induced poly(ADP-ribosyl)ation in SCs by DNA single-strand breaks, resulting in excessive activation of PARP (88–90). On the other hand, oxidative and nitrosative stress induces DNA damage by activating PARP (91). In streptozotocin-induced diabetic rats, the use of the PARP inhibitor 1,5-isoquinolinediol was accompanied by a decrease in poly(ADP-ribose), as well as a decrease in nitrotyrosine (NT) content in the sciatic nerve and neuro-vasculature and in superoxide content in the neuro-vasculature. This result suggests that PARP activation may lead to DPN through oxidative stress (92). An experiment found that combined treatment with two PARP inhibitors, FeTMPyP and 4-ANI, not only significantly attenuated oxidative nitrosative stress markers but also reduced excessive activation of PARP (93). Inhibition of PARP reduced the accumulation of NT, TNF-α, and 4-hydroxynonenal adduct accumulation in endothelial and SCs, spinal cord, and sensory neurons in the dorsal root ganglion (DRG) of diabetic peripheral nerves, attenuated diabetes-related oxidative and nitrosative stress, and alleviated peripheral nerve disorders (85, 94).

#### 3.2.6 Insulin pathway

Conventional thinking holds that insulin does not play a role in the direct regulation of central as well as peripheral nervous system function due to the insensitivity of neurons to insulin. However, there is growing evidence that insulin not only lowers blood glucose and thus is indirectly involved in the pathogenesis of DPN but also plays a direct role in the development of DPN as an important neurotrophic factor that supports peripheral nerves (95, 96). It has been shown that DRG expresses insulin receptors on the basal lamina, plasma membrane, and cytoplasmic processes of the SCs (97). While the dysfunction of the SCs plays an important role in the pathogenesis of DPN, insulin affects DPN by influencing SCs’ physiology.

A distinctive feature of DPN is demyelination as well as axonal damage, and insulin promotes axonal growth and improves demyelination as well as nerve conduction velocity (98). In spontaneously diabetic Wistar Bonn Kobori (WBN/Kob) rats, nerve conduction velocity was faster in the WBN plus insulin group compared to the WBN group, and axonal deformation and myelin expansion were improved in the sciatic and tibial nerves (99). A six-week treatment regimen of honey plus insulin improved sensory nerve conduction velocity in WBN/Kob rats (100). Insulin administration improves peripheral neuropathy in diabetic WBN/Kob rats. A study found that insulin reversed reduced lipoprotein lipase (LPL) expression in hyperglycemic SCs, improved demyelination caused by reduced LPL, and led to improved nerve morphology in the sciatic nerve (101). In the sciatic nerve, increased levels of myelin structural gene (P0) expression were accompanied by a significant increase in insulin receptor mRNA levels in SCs, while insulin also improved the levels of P0-related proteins as well as insulin receptor mRNA in SCs under hyperglycemic conditions (102). In a separate experiment, insulin receptor and insulin-like growth factor receptor 1 sphingolipids were found to be thinner in Chevron cell-specific knockout mice (103). These studies illustrated that insulin and its receptors, as well as insulin resistance, can affect myelin formation. The presence of insulin receptors on intrathecal neurons and the isolation of intrathecal insulin by intrathecal infusion of anti-insulin antibodies in non-diabetic rats produces slowed motor nerve conduction and axonal fiber atrophy (104). This study suggests that insulin itself also directly affects DPN, which may be related to the loss of insulin signaling. Insulin receptor signaling can promote axon growth through downstream signaling pathways such as the phosphatidylinositol 3-kinase (PI3K)-protein kinase B (Akt) signaling pathway (105–107). Activation of the Akt signaling pathway in SCs promotes their differentiation and also increases the formation of myelin sheaths (**Figure 3**) (108). In T1DM, DPN can be improved by the use of insulin (104, 109). In T2DM, insulin resistance arises due to decreased expression levels of insulin resistance, the altered phosphorylation status of insulin receptor substrate proteins, and impaired activation of axonal growth-related pathways, so providing insulin also fails to alter DPN (110–112). From the above, it is clear that insulin, insulin receptors, and insulin resistance, as participants in the PI3K-Akt signaling pathway, act in the DPN through the mechanism of impaired insulin signaling. This mechanism of insulin signaling may be related to maintaining the synthesis of key neuromodulatory proteins and peptides. More experiments are still needed to expand on the conditions related to signal production and to further investigate the role of related molecules in the insulin signaling pathway to discover new therapeutic pathways and approaches to target insulin signaling in DPN.

The figure shows the specific mechanisms of the PKC pathway, AGE pathway, insulin pathway, polyol pathway, and hexosamine pathway and reflects the relationships between each pathway. The key to the AGE pathway is the AGEs receptor. It can lead to chemokines and release pro-inflammatory markers. In high glucose environments, proteins and lipids are converted into glycogen end products that can bind to AGEs receptors, thereby mediating downstream pathways such as the PARP pathway to participate in oxidative stress or toxic glutamate accumulation and damage. The hexosamine and PKC pathways also cause nerve cell damage similarly. Glycolysis plays a crucial role in the mechanism of the three of them, as the intermediate product of glycolysis in the hexosamine pathway, fructose-6-phosphate, is ultimately converted into uridine diphosphate n-acetylglucosamine (UDP GlcNAc). UDP GlcNAk connects O-GlcNAc to serine and threonine residues of several transcription factors. Sp1 is one transcription factor that regulates PAI-1 and TGF-β Expression. PAI-1 can cause microvascular changes, and TGF-β can cause cell apoptosis and axonal damage through oxidative stress. Regarding the PKC pathway, the intermediate product 3-glyceraldehyde phosphate and the final product pyruvate are key factors in activating the PKC pathway. Pyruvate is used to prepare raw materials through the Krebs cycle as a PKC pathway. Glyceraldehyde-3-phosphate is converted into DAG and is involved in activating this pathway. The polyol pathway, on the other hand, results in significant conversion and increase of sorbitol due to high sugar environments. At the same time, it can also lead to an increase in fructose and an outflow of inositol. In neuropathy, excessive fructose and inositol efflux can cause certain damage to nerve cells. The figure also reflects the promoting function of insulin on axon formation.

### 3.3 Microvascular pathway

Currently, there is still controversy about how microvascular changes play a role in DPN. Inadequate blood and oxygen supply due to microvascular changes play a role in the mechanism of DPN. In the sciatic nerve, endothelial cell dysfunction due to reduced neural blood flow and intra-neural oxygen tension has been observed (113, 114). In addition, abnormal changes in the vasculature were observed in the peroneal nerve of DPN patients, and these abnormal changes included a decrease in vascular tight junction-associated proteins, thickening of the microvascular basement membrane within the nerve, proliferation, and swelling of the vascular endothelium, and degeneration of the pericytes. In turn, these abnormal changes will lead to vascular narrowing and affect blood flow, which will result in ischemia and hypoxia in peripheral nerve tissue (115–117). Hypoxia in the neural microenvironment will exacerbate oxidative stress and inflammation, leading to damage to SCs and neurons and ultimately causing nerve damage (118). However, changes in overall blood flow in the nervous system are not observed in all models, and nerve injury is not always due to altered blood flow. In a recent study, the first association between *in vivo* parameters of microvascular nerve perfusion and nerve conduction parameters and underlying clinical neuropathy scores was found in T2DM patients by using dynamic contrast-enhanced magnetic resonance neurography to study peripheral nerve microvascular permeability. Clinical and electrophysiological parameters of the tibial and peroneal nerves in T2DM patients were correlated with microvascular permeability and extravascular extracellular volume fraction but not with plasma volume fraction. Based on this association, it can be concluded that it is reduced microvascular permeability, not microvascular blood volume, that leads to nerve ischemia (119). As a result of the above controversy, some researchers have previously proposed the idea that changes in microvasculature do not lead to changes in overall blood flow but rather to disturbances in capillary blood flow patterns that affect blood and oxygen supply to the nerves. A concept related to this idea is capillary temporal heterogeneity (CTH), where a mild elevation of CTH leads to poor oxygen extraction and improves this state by shifting to a congested state. When CTH is further elevated, it leads to endothelial dysfunction and low tissue oxygen tension, resulting in impaired neurological function (115). Since it is still difficult to link microvascular changes to the initial mechanism of DPN production, it has been suggested that microvascular changes are not an initiating factor but rather contribute to the later development of DPN (120). DPN, when the body is at rest or during exercise, can lead to microvascular disorders through altered endothelial barriers and neurogenic mechanisms (121, 122). The question of whether microvascular changes appear before the development of DPN or act later in the development of DPN is still under investigation. In conclusion, although controversy about the role of microvascular still exists, microvascular changes should play a role in the development of DPN, which requires more experiments to determine the role of microvascular.

### 3.4 Other pathways

These above-mentioned pathways are more studied. Recently, some new pathways have been discovered, which are likely to be new targets for the treatment of DPN in the future. These new pathways include the Wnt pathway, MAPK pathway, mTOR pathway, and thyrotropin (TSH) pathway.

#### 3.4.1 Wnt/β-catenin pathway

Wnt gene is a gene family consisting of at least 19 genes. Related to the Wnt pathway, β-catenin is involved in the transcription of the Wnt pathway and promotes cell adhesion. The Wnt/β-catenin pathway is activated through the binding of Wnt ligands to receptors. When the Wnt/β-catenin pathway is activated, the Wnt protein is transferred to the Golgi apparatus and binds to the transmembrane protein Wls secreted by Wnt. Subsequently, the Wnt ligand was transferred to the cell membrane. This Wnt ligand can bind to the receptors of the frizzled protein family to activate various downstream signaling pathways (123). The Wnt protein located outside the cell can activate three intracellular transduction cascades: the canonical Wnt/β-catenin pathway, the non-canonical planar cell polarity pathway, and the Wnt/Ca^2+^ pathway (124).

The Wnt/β-catenin signaling pathway plays an important regulatory role in cell proliferation, differentiation, development, and metabolism (125). Research has shown that the Wnt/β-catenin signaling pathway is also related to demyelination. When this pathway is activated, the amount of intracellular free β-catenin increases and enters the nucleus. This is related to the downstream Akt signaling pathway. It induces the immortalization of SCs and participates in high glucose-promoting apoptosis of SCs (30, 126, 127). Resham et al. showed that the sciatic nerve of diabetes neuropathy rats showed Wnt pathway protein, namely β-catenin, c-myc, and matrix metallopeptidase 2 increased (128). These all indicate that Wnt/β-catenin pathway plays a significant role in DPN (**Figure 3**). Although many pieces of research have shown that the pathways can destroy nerve cells, a study on human placenta-derived mesenchymal stem cells (PMSCs) ameliorating diabetic neuropathy via Wnt signaling pathway shows that Wnt pathway can promote the improvement of PMSCs on diabetes peripheral neuropathy and promote nerve cell regeneration (129).

Moreover, it has also been proven to regulate the function of pancreatic organs and play a role in pancreatic beta cells and glucose-stimulated insulin secretion (130). The Wnt pathway may also play a role in more aspects, but the role and mechanism of this pathway still need to be studied.

#### 3.4.2 MAPK pathway

A crucial signaling system that controls several cellular activities, including proliferation, differentiation, apoptosis, and stress response, is the mitogen-activated protein kinase (MAPK) cascade. It can transduce extracellular stimuli into cells. Research has shown that it is related to mitochondrial failure caused by metabolic disorders (131, 132).

MAPK is a serine/threonine protein kinase family that includes three subtypes: p38 MAPK, extracellular signal-regulated protein kinase (ERK1/2), and c-Jun N-terminal kinase/stress-activated protein kinase (SAPK/JNK). The oxidative stress and extracellular stimuli, such as Ca^2+^ generated by upstream pathways, can stimulate the phosphorylation of MAPKs and activate the MAPK pathway. In addition to acting as a downstream pathway, hyperglycemia may directly cause phosphorylation of MAPKs (**Figure 3**) (132, 133). A study on peripheral neuropathy caused by paclitaxel showed that MAPK signaling pathways such as JNK, ERK1/2, and nuclear factors- κB played a major role in it (134).

Among them, p38 MAPK is involved in glucose and lipid metabolism. The p38 subtype phosphorylates to activate enzymes involved in glucose and lipid metabolism. Some studies have shown that p38 activation is found in the dorsal root ganglia of diabetes rats (132, 135). These all demonstrate the important role of the MAPK pathway in neuropathy.

However, there are also studies indicating that the MAPK signaling pathway is also related to neuroprotection and nerve regeneration (136). The diversity of MAPK pathway functions still needs to be explored, and how to make good use of this mechanism is the direction that we should strive for.

#### 3.4.3 mTOR pathway

As an ATP receptor, the mammalian target of rapamycin (mTOR) regulates cell growth and proliferation depending on nutrient and energy status. mTOR is one of the downstream targets of AMPK and is also able to interact with AMPK (137). In the pathogenesis of DPN, mTOR is mainly involved through three pathways: autophagy and apoptosis of SCs, neurotrophic factors, and myelin formation (**Figure 3**). A certain degree of autophagy in SCs can play a neuroprotective role (138), but sustained autophagy is closely related to cell death, and autophagy in SC is closely related to the development of DPN with impact (139).

A study found that *Lycium barbarum* polysaccharide promotes autophagy in SCs by inhibiting the activation of the mTOR/p70S6K pathway. The activation of mTOR can upregulate autophagy to some extent, thus playing a protective role in DPN (140). Another study on astragaloside IV found that enhancing autophagy by inhibiting activation of the PI155K/Akt/mTOR signaling pathway can alleviate apoptosis-induced myelin damage in DPN by SCs (141). Although inhibition of mTOR can attenuate neurological damage in DPN by promoting autophagy, not all mTOR inhibitions are protective. RSC96 cells cultured in a high glucose medium can be used to mimic SCs in DPN mice. A study with RSC96 cells found that in the HG situation, the Akt/mTOR signaling pathway was inhibited, and autophagy and apoptosis were increased in RSC96 cells. However, muscarinic ketones ameliorated this situation, thereby attenuating DPN (142). mTOR kinase is present in two different multiprotein complexes, mTORC1 and mTORC2 (143), and phosphorylated mTOR was reduced in the sciatic nerve of diabetic mice, with increased apoptosis in SCs. Also, in RSC96 cells, inhibition of mTORC1 promoted apoptosis by silencing PARTOR or RICTOP (144). These studies suggest that inhibition of the mTOR pathway causes sustained autophagy and apoptosis in SCs, which in turn affects DPN development and progression.

Neurotrophic factors secreted by SCs play an important role in maintaining the normal structure and function of peripheral nerves (145, 146). mTOR, as an upstream signaling molecule of DNA methyltransferase 1 (DNMT1), influences the secretion of neurotrophic factors in SCs by regulating DNMT1. Zhang et al. found that in RSC96 cells, hyperglycemia downregulated brain-derived neurotrophic factor (BDNF) by inhibiting the Akt/mTOR pathway led to enhanced expression of DNMT1 and thus downregulated BDNF in RSC96 cells. In contrast, BDNF deficiency in SCs plays an important role in the development of DPN (147). In addition, mTOR may also be involved in the mechanism of DPN development by affecting myelin and axons through the regulation of lipid metabolism in SCs. mTORC1 activation has different effects in different periods of SCs. Some researchers found that mTORC1 activity is downregulated in developing SC during normal neuro myelin formation, but persistently elevated mTORC1 in differentiated SC elevation manifests as excessive myelination in late adulthood (143), leading to abnormal axon production (148). It has been found that insulin resistance affects myelin enhancement or hypomyelination in SCs by affecting the mTOR pathway, which leads to altered axons in the peripheral nervous system (103).

mTOR has attracted the attention of researchers as a relatively new mechanism in DPN. However, it is still full of unknowns about how mTOR plays a role in lipid and energy metabolism, insulin resistance, and cellular autophagy, and many upstream influences on mTOR are still unexplored areas that need further studies to come.

#### 3.4.4 TSH pathway

Clinical and subclinical hypothyroidism (SCH) in patients with diabetes mellitus is quite common in patients with DPN and is strongly associated with the severity of DPN (149). In several studies investigating the relationship between TSH and DPN in patients with T2DM, TSH levels were found to be positively correlated with DPN (150–152). Also, in an investigation on hypothyroid women, TSH levels were elevated in patients newly diagnosed with diabetic neuropathy (153). In a study as early as 1999, TSH was found to have acute effects on DPN, and nerve conduction velocity (NCV) was improved in streptozotocin-diabetic rats treated with TSH (154). In a recent study, Fan et al. found abnormalities in glycolipid metabolism in a SCH-T2DM mouse model while also observing that TSH behaved consistently with apoptosis-associated proteins in SCs. In subsequent *in vitro* experiments, oxidative stress, as well as mitochondrial damage, was found to be increased by TSH in HG and PA-conditioned RSC96 cells. Palmitoylation of thyrotropin receptor (TSH-R) increases apoptosis in RSC96 cells, and this is reversed after TSHR knockdown or inhibition of TSHR palmitoylation (155). There are few studies on the role of TSH in the pathogenesis of DPN, and the relevant mechanistic studies only refer to aspects concerning SCs and do not involve studies concerning neurons. The downstream pathways of TSH are still unknown, and it is not clear whether they intersect with other mechanistic pathways. Further studies on the downstream signaling pathways of TSH should be conducted in the future to discover better targets for the treatment of DPN.

This figure reflects other avenues that need to be studied in this article, including the Wnt/β-catenin pathway, MAPK pathway, TSH pathway, and mTOR pathway. Wnt protein is modified in the endoplasmic reticulum and further transported out of cells through vesicles. This is the key to the Wnt pathway. β-Catenin plays a role in triggering downstream pathways. The TSH pathway may also be related to MAPK, and abnormalities in the TSH pathway can lead to oxidative stress and excessive production of reactive oxygen species, leading to damage to the MAPK pathway. TSH is also related to calcium ion concentration. The mTOR pathway plays a more complex role. Akt is activated by extracellular signals through PI155K, while Akt is inhibited by hyperglycemic Akt, leading to mTOR activation. Silence of PARTOR and RICTOR leads to inhibition of mTORC1/mTORC2. AMPK inhibits mTORC1 by silencing PARTOR. The activation of mTOR affects the myelin sheath and axons, inhibits cell autophagy, and also affects BDNF by inhibiting the expression of DNMT1. These three paths of mTOR ultimately affect DPN.

## 4 Diagnosis and treatment

### 4.1 Screening and diagnosis

The screening of DPN includes detailed medical history collection and five basic sensory tests, including ankle reflex, vibration sensation, pressure sensation, acupuncture pain sensation, and temperature sensation (10-g Semmes-Weinstein monofilament for light touch, Tiptherm rod for temperature, calibrated Rydel Seiffer tuning fork for vibration, pin-prick for pain) (156).

In general, the diagnosis of DSPN is based on clinical signs and symptoms, which can be standardized according to various quantitative criteria (like Michigan Neuropathy Screening Instrument) (157), the Neuropathy Symptom Score (158) or Total Symptom Score (159) for neuropathic symptoms and the Neuropathy Disability Score for neuropathic signs (158). Only when the symptoms are not typical further nerve conduction study (NCS), quantitative sensory testing, and intraepidermal nerve fiber density will be performed. In addition, quantitative measurement of tibial nerve T2 values using magnetic resonance imaging has also been shown to be a non-invasive and reliable method of diagnosing and monitoring the progression of DPN (160).

### 4.2 Strict blood sugar control

As a direct cause of DPN, controlling blood sugar levels is of great significance in the subsequent treatment process of DPN. It has been demonstrated that the incidence of peripheral neuropathy increases with worsening blood glucose status and is approximately five times more common in patients with confirmed diabetes than in those with normal blood glucose (12). Although aggressive glycemic control can significantly reduce the risk and rate of progression of DPN in T1DM, this approach has limited benefit in T2DM, mainly in terms of improvements in NCS outcomes and vibration perception thresholds (161, 162). Therefore, in addition to blood glucose, in recent years, researchers have expanded their studies and started to explore the association between metabolic syndrome (MetS) and DPN. It has been shown that MetS and several of its components increase the risk of neuropathy in patients with established T1DM and T2DM (161). For example, obesity is considered an important metabolic driver of DPN, and statistical studies based on populations from different regions of the world have confirmed that obesity is a potential cause of peripheral neuropathy in non-diabetic obese patients (12, 163–165). This requires the clinician to provide appropriate exercise and diet control while maintaining stable control of the patient’s blood glucose to effectively intervene with the adverse effects of metabolic factors on DPN.

### 4.3 Medication

**Figure 4:**
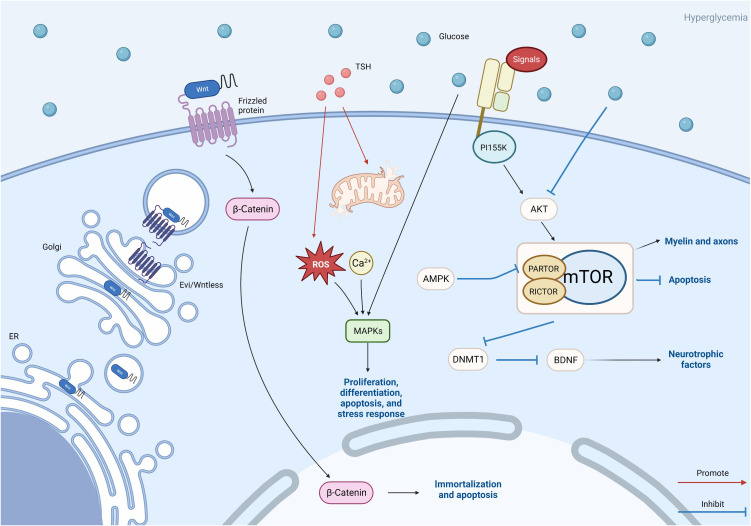
Pattern diagram of other pathways.

**Figure 5:**
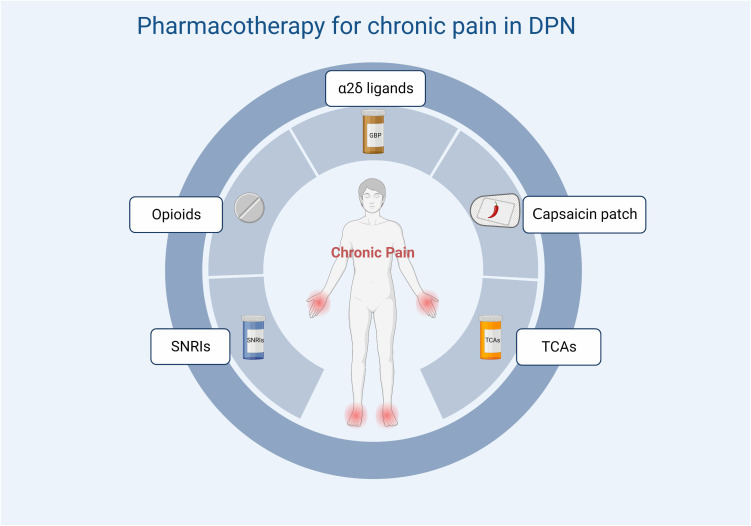
Treatment options for chronic pain in DPN. SNRIs, serotonin and norepinephrine reuptake inhibitors; TCAs, tricyclic antidepressants.

A variety of drugs are currently used in clinical practice for the treatment of DPN and can be classified according to their action as symptom-ameliorating drugs and therapeutic drugs that target pathogenesis. There are no drugs available to reverse the progression of DPN. For chronic pain, which is often associated with DPN, current clinical options include anticonvulsants (pregabalin, gabapentin), tricyclic antidepressants (amitriptyline), and serotonin-noradrenaline reuptake inhibitors (duloxetine). The first-line drugs most often recommended for the treatment of painful DSPN are α2δ ligands (gabapentin and pregabalin). Tricyclic antidepressants have been restricted because of their potential cholinergic adverse effects, especially in older patients. For opioids, although studies have also demonstrated their efficacy in neuropathic pain associated with DPN (17, 166), they should not be used routinely due to limited efficacy, long-term safety concerns, and potential for abuse. Topical analgesic therapy can be a new option for pain that cannot be effectively managed with the above-mentioned medications. An alternative treatment is the capsaicin 8% patch, which contains 179 mg or 8% capsaicin weight for weight. It has been shown to be well tolerated and provides effective pain relief for a variety of types of peripheral neuropathic pain (**Figure 4**) (167). Other than this, the 5% lidocaine patch used to treat postherpetic neuralgia also appears to be available for the treatment of painful DSPN. Although this usage has not been authorized, Results from a large open-label controlled study suggest that the lidocaine plaster could be at least as effective as systemic pregabalin in the treatment of painful diabetic polyneuropathy (168) **Figure 5**.

Other drugs used to improve symptoms include drugs to improve microcirculation (prostaglandins and prostaglandin analogues, hexoketocin, pancreatic kininogenase, Bactrim), neurotrophic drugs (methylcobalamin), drugs to improve cellular energy metabolism, drugs to combat oxidative stress (alpha-lipoic acid), inhibitors of aldose reductase activity (epalrestat), angiotensin-converting enzyme inhibitors. Many studies have reported that these drugs, alone or in combination with other drugs, can promote peripheral nerve regeneration and improve clinical symptoms in patients with DPN (169), but the magnitude of their benefits remains controversial. Although a wide range of drugs are available, there is still a lack of specific drugs and treatment options for DPN due to its complex pathogenesis, diverse clinical manifestations, and immature staging. This has led to many current drug regimens being relatively conservative, limiting doses to avoid serious side effects and ultimately compromising treatment outcomes. As a result, several studies are currently exploring combination drug regimens for DPN intending to control the symptoms and progression of DPN while minimizing serious adverse effects and improving patient compliance.

Another study compared the comparative safety and tolerability of duloxetine vs. pregabalin vs. duloxetine plus gabapentin in patients with diabetic peripheral neuropathic pain, suggesting that the duloxetine plus gabapentin regimen is generally safe and well-tolerated (170).

Lipoic acid (LA) is a member of the vitamin B family, which plays a critical role in eliminating free radicals that can accelerate aging and cause diseases. A meta-analysis has shown that α-lipoic acid (ALA) supplementation significantly reduced insulin and homeostatic model assessment of insulin resistance (171). In addition to single-agent use, ALA has shown significant benefits and safety in combination with other drugs. A meta-analysis from China evaluates the efficacy of ALA plus epalrestat combination therapy in the treatment of DPN. The results showed that the combination of ALA plus epalrestat clearly improved clinical efficacy and accelerated nerve conduction compared to ALA or epalrestat monotherapy (172). Another Meta-analysis shows treatment with ALA plus methylcobalamin (MC) once a day for 2–4 weeks resulted in better improvement in neuropathic symptoms and NCVs compared with the administration of MC alone. Moreover, compared with MC alone, LA–MC combination therapy was not associated with more severe adverse events in patients with DPN (173).

In addition to ALA, clinical trials have shown that the combination of gabapentin (GBP) and vitamin B1-B12 creates a synergistic effect due to their anti-allodynic and anti-hyperalgesic effect. Pain intensity reduction is achieved with 50% of the minimum required GBP dose alone (800 to 1600 mg/d) in the GBP/B1/B12 group. Furthermore, less vertigo and dizziness occurrence were also observed in the GBP/B1/B12 group (174).

Prostaglandin E1 is primarily used clinically to relax blood vessels, reduce blood viscosity, and inhibit platelet aggregation. As a drug that improves microcirculation, its clinical use in the treatment of DPN has been confirmed in several studies. The effectiveness and safety of its use in combination with other drugs have been analyzed in several studies. A meta-analysis of 31 randomized controlled trials (RCTs) with 2,676 participants evaluated the efficacy and safety of prostaglandin E1 (PGE1) in combination with LA for the treatment of DPN compared with PGE1 or LA monotherapy. The results show that the clinical efficacy of PGE1 plus LA combination therapy was significantly better than monotherapy (p < 0.00001, RR = 1.32, 95% CI=1.26 to 1.38) (175). Another Meta-analysis of 16 RCTs with 1136 participants showed that the clinical efficacy of methylcobalamin plus PGE1 combination therapy was significantly better than PGE1 monotherapy (fifteen trials; RR 1.25, 95% CI 1.18-1.32, P<0.012 = 27%) (176).

The results of such studies on combination therapy ultimately suggest that, as DPN is a complex diabetic complication caused by chronic hyperglycemia and associated with multiple factors such as metabolic disorders, microvascular disease, neurotrophic factor deficiencies, and oxidative stress, its treatment must be based on a combination of pathogenic mechanisms in order to achieve a satisfactory outcome. For the present, despite the wide range of drugs available, the evidence on their long-term effectiveness and the effectiveness of combination therapy remains incomplete, and feedback on these drugs is inconsistent among patients. Well-designed multicenter RCTs are required to confirm these findings.

### 4.4 Non-pharmacological treatments

As the efficacy of existing pharmacological treatments for DSPN is equivocal, non-pharmacological treatments are also widely used clinically as an adjunct to pharmacological treatments, albeit with a lower level of evidence (177). These treatments include psychological support, acupuncture, physiotherapy, and transcutaneous electrical nerve or muscle stimulation. Another promising non-pharmacological treatment for DPN is spinal cord stimulation (SCS), which has been used for over 40 years to treat localized chronic refractory neuropathic pain in the limbs and trunk, and with continuing advances in technology, its efficacy and safety have been further demonstrated. A study has shown that patients with painful diabetic neuropathy refractory to the best available treatments can be safely and effectively treated with high-frequency (10kHz) SCS, and follow-up of this study population over 24 months has demonstrated the potential durability of this treatment beyond 6 months (178).

## 5 Discussion

This review describes and summarizes the problems related to peripheral neuropathy caused by diabetes. What we are most concerned about is the mechanism of the disease, as this is the core issue of treating and researching the disease. How hyperglycemia, dyslipidemia, and insulin resistance lead to inflammation, oxidative stress, and other changes leading to nerve cell damage has not been fully explored. The specific molecular mechanism of the interaction between them is a goal that we should further study. The relationship between different pathways, such as antagonism or synergy, and the actual molecular mechanisms that cause damage to nerve cells still need to be discussed. For example, the interaction between the polyol pathway and PKC pathway through intermediate products in the glycolysis process. The PKC pathway, polyol pathway, advanced glycation end products pathway, hemoglobin pathway, PARP pathway, etc., have been extensively studied, but there are still unknown or potential ways of action that have not been noticed. For example, the PKC pathway and polyol pathway often participate in other pathways to synergize and exacerbate oxidative stress. Or there are more molecular mechanisms of neuronal damage and death caused by diabetes-related factors such as hyperglycemia, dyslipidemia, insulin resistance, and so on, waiting to be explored. The interaction between pathways should become the direction of future research. In addition to the aforementioned pathways, we also discussed some relatively new pathogenic pathways, Wnt/β-catenin pathway, MAPK pathway, mTOR pathway, and TSH pathway, either as downstream pathways, lead to oxidative stress and metabolic disorders to indirectly lead to nerve cell damage and death, or directly affect neural cells to cause damage.

Due to the lack of relevant research and investigation, the mechanism of diabetes symptoms, such as neuropathy, blood glucose, and dyslipidemia, has not been determined. We have not yet gained a deep understanding of this field. These pathways have great potential in terms of treatment and medication. According to the current research, it can be determined that the mechanism of peripheral neuropathy caused by T1DM and T2DM is not the same, but the specific mechanism difference is not yet clear. Many experiments show that the therapeutic effect of certain substances varies with the type of diabetes. From the perspective of disease treatment, this blind spot may be able to clarify the effect of drugs to better treat DPN.

It should also be mentioned that many studies have shown that the above mechanism of DPN is not unique. The same pathway can mediate both damage to nerve cells and the protective and regenerative effects of nerve cells. That is to say, many molecular mechanisms have hermaphroditism. It is a challenge to make good use of these molecular mechanisms to selectively protect and regenerate them and avoid causing damage to nerve cells. From the perspective of treatment and medication, this is also an important target for drug efficacy and can become an important part of the research.

## Author contributions

JZ: Writing – original draft. ZH: Writing – original draft. YLu: Writing – original draft. YLi: Investigation, Visualization, Writing – original draft. WL: Methodology, Visualization, Writing – original draft. XD: Funding acquisition, Writing – review & editing. SP: Conceptualization, Funding acquisition, Project administration, Writing – review & editing. ZL: Conceptualization, Funding acquisition, Project administration, Writing – review & editing. JH: Conceptualization, Writing – review & editing.

## References

[1] Hanewinckel R van Oijen M Ikram MA van Doorn PA. The epidemiology and risk factors of chronic polyneuropathy. Eur J Epidemiol (2016) 31(1):5–20. doi: 10.1007/s10654-015-0094-6 26700499 PMC4756033

[2] Gordois A Scuffham P Shearer A Oglesby A Tobian JA. The health care costs of diabetic peripheral neuropathy in the US. Diabetes Care (2003) 26(6):1790–5. doi: 10.2337/diacare.26.6.1790 12766111

[3] Sloan G Shillo P Selvarajah D Wu J Wilkinson ID Tracey I. A new look at painful diabetic neuropathy. Diabetes Res Clin Practice (2018) 144:177–91. doi: 10.1016/j.diabres.2018.08.020 30201394

[4] Jeffcoate WJ Vileikyte L Boyko EJ Armstrong DG Boulton AJM. Current challenges and opportunities in the prevention and management of diabetic foot ulcers. Diabetes Care (2018) 41(4):645–52. doi: 10.2337/dc17-1836 29559450

[5] Boulton AJM Armstrong DG Kirsner RS Attinger CE Lavery LA Lipsky BA. Diagnosis and management of diabetic foot complications. Compendia (2018) 2018(2). doi: 10.2337/db20182-1 30958663

[6] Miller JW. Proton pump inhibitors, H2-receptor antagonists, metformin, and vitamin B-12 deficiency: clinical implications. Adv Nutr (2018) 9(4):511s–8s. doi: 10.1093/advances/nmy023 PMC6054240 30032223

[7] Bell DSH. Metformin-induced vitamin B12 deficiency can cause or worsen distal symmetrical, autonomic and cardiac neuropathy in the patient with diabetes. Diabetes Obes Metab (2022) 24(8):1423–8. doi: 10.1111/dom.14734 35491956

[8] Li Y Teng D Shi X Qin G Qin Y Quan H. Prevalence of diabetes recorded in mainland China using 2018 diagnostic criteria from the American Diabetes Association: national cross sectional study. Br Med J (2020) 369:m997. doi: 10.1136/bmj.m997 32345662 PMC7186854

[9] Gwathmey KG Pearson KT. Diagnosis and management of sensory polyneuropathy. Br Med J (2019) 365:l1108. doi: 10.1136/bmj.l1108 31068323

[10] Badiu C. Williams textbook of endocrinology - 14th revised edition. Acta Endocrinologica (Bucharest) (2019) 15(3):416–. doi: 10.4183/aeb.2019.416

[11] Shabeeb D Najafi M Hasanzadeh G Hadian MR Musa AE Shirazi A. Electrophysiological measurements of diabetic peripheral neuropathy: A systematic review. Diabetes Metab Syndrome: Clin Res Rev (2018) 12(4):591–600. doi: 10.1016/j.dsx.2018.03.026 29610062

[12] Callaghan BC Gao L Li Y Zhou X Reynolds E Banerjee M. Diabetes and obesity are the main metabolic drivers of peripheral neuropathy. Ann Clin Trans Neurology (2018) 5(4):397–405. doi: 10.1002/acn3.531 PMC5899909 29687018

[13] Kirchmair R Weisman A Bril V Ngo M Lovblom LE Halpern EM. Identification and prediction of diabetic sensorimotor polyneuropathy using individual and simple combinations of nerve conduction study parameters. PloS One (2013) 8(3):e58783. doi: 10.1371/journal.pone.0058783 23533591 PMC3606395

[14] Pan Q Li Q Deng W Zhao D Qi L Huang W. Prevalence of and risk factors for peripheral neuropathy in chinese patients with diabetes: A multicenter cross-sectional study. Front Endocrinology (2018) 9:617. doi: 10.3389/fendo.2018.00617 PMC6230581 30455667

[15] Ziegler D Papanas N Vinik AI Shaw JE. Epidemiology of polyneuropathy in diabetes and prediabetes. Handb Clin Neurology (2014) 126:3–22. doi: 10.1016/B978-0-444-53480-4.00001-1 25410210

[16] Pan Q Li Q Deng W Zhao D Qi L Huang W. Prevalence and diagnosis of diabetic cardiovascular autonomic neuropathy in beijing, China: A retrospective multicenter clinical study. Front Neurosci (2019) 13:1144. doi: 10.3389/fnins.2019.01144 31708736 PMC6823192

[17] Sloan G Selvarajah D Tesfaye S. Pathogenesis, diagnosis and clinical management of diabetic sensorimotor peripheral neuropathy. Nat Reviews: Endocrinology (2021) 17(7):400–20. doi: 10.1038/s41574-021-00496-z 34050323

[18] Enders J Elliott D Wright D. Emerging nonpharmacologic interventions to treat diabetic peripheral neuropathy. Antioxid Redox Signal (2022) 38(13-15):989ߝ1000. doi: 10.1089/ars.2022.0158 PMC10402707 36503268

[19] Carstens MH Quintana FJ Calderwood ST Sevilla JP Ríos AB Rivera CM. Treatment of chronic diabetic foot ulcers with adipose-derived stromal vascular fraction cell injections: Safety and evidence of efficacy at 1 year. Stem Cells Trans Med (2021) 10(8):1138–47. doi: 10.1002/sctm.20-0497 PMC8284780 33826245

[20] Feldman EL Callaghan BC Pop-Busui R Zochodne DW Wright DE Bennett DL. Diabetic neuropathy. Nat Rev Dis Primers (2019) 5(1):41. doi: 10.1038/s41572-019-0092-1 31197153

[21] Li J Guan R Pan L. Mechanism of Schwann cells in diabetic peripheral neuropathy: A review. Med (Baltimore) (2023) 102(1):e32653. doi: 10.1097/MD.0000000000032653 PMC9829292 36607875

[22] Elafros MA Andersen H Bennett DL Savelieff MG Viswanathan V Callaghan BC. Towards prevention of diabetic peripheral neuropathy: clinical presentation, pathogenesis, and new treatments. Lancet Neurology (2022) 21(10):922–36. doi: 10.1016/S1474-4422(22)00188-0 PMC10112836 36115364

[23] Lin T Gargya A Singh H Sivanesan E Gulati A. Mechanism of peripheral nerve stimulation in chronic pain. Pain Med (2020) 21(Suppl 1):S6–s12. doi: 10.1093/pm/pnaa164 32804230 PMC7828608

[24] Previtali SC. Peripheral nerve development and the pathogenesis of peripheral neuropathy: the sorting point. Neurotherapeutics (2021) 18(4):2156–68. doi: 10.1007/s13311-021-01080-z PMC8804061 34244926

[25] Hoke A Cerri F Fisgin A Riva N Quattrini A. Normal structure and pathological features in peripheral neuropathies. J Peripheral Nervous System (2021) 26 Suppl 2:S11–s20. doi: 10.1111/jns.12462 34768313

[26] Bosch-Queralt M Fledrich R Stassart RM. Schwann cell functions in peripheral nerve development and repair. Neurobiol Disease (2023) 176:105952. doi: 10.1016/j.nbd.2022.105952 36493976

[27] Salzer JL. Schwann cell myelination. Cold Spring Harbor Perspect Biol (2015) 7(8):a020529. doi: 10.1101/cshperspect.a020529 PMC4526746 26054742

[28] Monje M. Myelin plasticity and nervous system function. Annu Rev Neurosci (2018) 41:61–76. doi: 10.1146/annurev-neuro-080317-061853 29986163

[29] Du W Wang N Li F Jia K An J Liu Y. STAT3 phosphorylation mediates high glucose-impaired cell autophagy in an HDAC1-dependent and -independent manner in Schwann cells of diabetic peripheral neuropathy. FASEB J (2019) 33(7):8008–21. doi: 10.1096/fj.201900127R 30913399

[30] Liu YP Shao SJ Guo HD. Schwann cells apoptosis is induced by high glucose in diabetic peripheral neuropathy. Life Sci (2020) 248:117459. doi: 10.1016/j.lfs.2020.117459 32092332

[31] Cernea S Raz I. Management of diabetic neuropathy. Metabolism: Clin Experimental (2021) 123:154867. doi: 10.1016/j.metabol.2021.154867 34411554

[32] Shen J. Plasticity of the central nervous system involving peripheral nerve transfer. Neural Plasticity (2022) 2022:5345269. doi: 10.1155/2022/5345269 35342394 PMC8956439

[33] Feldman EL Nave KA Jensen TS Bennett DLH. New horizons in diabetic neuropathy: mechanisms, bioenergetics, and pain. Neuron (2017) 93(6):1296–313. doi: 10.1016/j.neuron.2017.02.005 PMC5400015 28334605

[34] DiAntonio A. Axon degeneration: mechanistic insights lead to therapeutic opportunities for the prevention and treatment of peripheral neuropathy. Int Assoc Study Pain (2019) 160 (Suppl 1):S17–s22. doi: 10.1097/j.pain.0000000000001528 PMC6481657 31008845

[35] Modrak M Talukder MAH Gurgenashvili K Noble M Elfar JC. Peripheral nerve injury and myelination: Potential therapeutic strategies. J Neurosci Res (2020) 98(5):780–95. doi: 10.1002/jnr.24538 PMC7072007 31608497

[36] Kamiya H Murakawa Y Zhang W Sima AA. Unmyelinated fiber sensory neuropathy differs in type 1 and type 2 diabetes. Diabetes Metab Res Review (2005) 21(5):448–58. doi: 10.1002/dmrr.541 15747389

[37] Sima AA Kamiya H. Diabetic neuropathy differs in type 1 and type 2 diabetes. Ann New York Acad Sci (2006) 1084:235–49. doi: 10.1196/annals.1372.004 17151305

[38] Hur J O’Brien PD Nair V Hinder LM McGregor BA Jagadish HV. Transcriptional networks of murine diabetic peripheral neuropathy and nephropathy: common and distinct gene expression patterns. Diabetologia (2016) 59(6):1297–306. doi: 10.1007/s00125-016-3913-8 PMC4862920 27000313

[39] Gu Y Qiu ZL Liu DZ Sun GL Guan YC Hei ZQ. Differential gene expression profiling of the sciatic nerve in type 1 and type 2 diabetic mice. Biomed Rep (2018) 9(4):291–304. doi: 10.3892/br.2018.1135 30233781 PMC6142038

[40] Mizukami H Osonoi S. Pathogenesis and molecular treatment strategies of diabetic neuropathy collateral glucose-utilizing pathways in diabetic polyneuropathy. Int J Mol Sci (2020) 22(1):94. doi: 10.3390/ijms22010094 33374137 PMC7796340

[41] Boland BB Rhodes CJ Grimsby JS. The dynamic plasticity of insulin production in β-cells. Mol Metab (2017) 6(9):958–73. doi: 10.1016/j.molmet.2017.04.010 PMC5605729 28951821

[42] Ryan DG O’Neill LAJ. Krebs cycle reborn in macrophage immunometabolism. Annu Rev Immunol (2020) 38:289–313. doi: 10.1146/annurev-immunol-081619-104850 31986069

[43] Limaye AJ Bendzunas GN Kennedy EJ. Targeted disruption of PKC from AKAP signaling complexes. RSC Chem Biol (2021) 2(4):1227–31. doi: 10.1039/D1CB00106J PMC8341804 34458835

[44] Megha R Farooq U Lopez PP. Stress-induced gastritis. StatPearls. Treasure Island (FL: ): StatPearls PublishingCopyright © 2023, StatPearls Publishing LLC (2023). 29763101

[45] Huang SK Lu CW Lin TY Wang SJ. Neuroprotective role of the B vitamins in the modulation of the central glutamatergic neurotransmission. CNS Neurological Disord Drug Targets (2022) 21(4):292–301. doi: 10.2174/1871527320666210902165739 34477538

[46] Dal-Cim T Poluceno GG Lanznaster D de Oliveira KA Nedel CB Tasca CI. Guanosine prevents oxidative damage and glutamate uptake impairment induced by oxygen/glucose deprivation in cortical astrocyte cultures: involvement of A(1) and A(2A) adenosine receptors and PI3K, MEK, and PKC pathways. Purinergic Signal (2019) 15(4):465–76. doi: 10.1007/s11302-019-09679-w PMC6923312 31520282

[47] Asiri MMH Engelsman S Eijkelkamp N Höppener JWM. Amyloid proteins and peripheral neuropathy. Cells (2020) 9(6):1553. doi: 10.3390/cells9061553 32604774 PMC7349787

[48] Vastani N Guenther F Gentry C Austin AL King AJ Bevan S. Impaired nociception in the diabetic ins2(+/akita) mouse. Diabetes (2018) 67(8):1650–62. doi: 10.2337/db17-1306 29875100

[49] Chau DD Li W Chan WWR Sun JK Zhai Y Chow HM. Insulin stimulates atypical protein kinase C-mediated phosphorylation of the neuronal adaptor FE65 to potentiate neurite outgrowth by activating ARF6-Rac1 signaling. FASEB J (2022) 36(11):e22594. doi: 10.1096/fj.202200757R 36250347

[50] Kawano T Inokuchi J Eto M Murata M Kang JH. Activators and inhibitors of protein kinase C (PKC): their applications in clinical trials. Pharmaceutics (2021) 13(11):1748. doi: 10.3390/pharmaceutics13111748 34834162 PMC8621927

[51] Kobayashi M Zochodne DW. Diabetic polyneuropathy: Bridging the translational gap. J Peripheral Nervous System (2020) 25(2):66–75. doi: 10.1111/jns.12392 32573914

[52] Elmazoglu Z Prnova MS Stefek M Ceylan AF Aschner M Rangel-López E. Protective effects of novel substituted triazinoindole inhibitors of aldose reductase and epalrestat in neuron-like PC12 cells and BV2 rodent microglial cells exposed to toxic models of oxidative stress: comparison with the pyridoindole antioxidant stobadine. Neurotoxicity Res (2021) 39(3):588–97. doi: 10.1007/s12640-021-00349-7 33713301

[53] Papachristoforou E Lambadiari V Maratou E Makrilakis K. Association of glycemic indices (Hyperglycemia, glucose variability, and hypoglycemia) with oxidative stress and diabetic complications. J Diabetes Res (2020) 2020:7489795. doi: 10.1155/2020/7489795 33123598 PMC7585656

[54] Niimi N Yako H Takaku S Chung SK Sango K. Aldose reductase and the polyol pathway in schwann cells: old and new problems. Int J Mol Sci (2021) 22(3):1031. doi: 10.3390/ijms22031031 33494154 PMC7864348

[55] Langer HT Afzal S Kempa S Spuler S. Nerve damage induced skeletal muscle atrophy is associated with increased accumulation of intramuscular glucose and polyol pathway intermediates. Sci Rep (2020) 10(1):1908. doi: 10.1038/s41598-020-58213-1 32024865 PMC7002415

[56] Freeman OJ Unwin RD Dowsey AW Begley P Ali S Hollywood KA. Metabolic dysfunction is restricted to the sciatic nerve in experimental diabetic neuropathy. Diabetes (2016) 65(1):228–38. doi: 10.2337/db15-0835 26470786

[57] Daniels LJ Annandale M Koutsifeli P Li X Bussey CT van Hout I. Elevated myocardial fructose and sorbitol levels are associated with diastolic dysfunction in diabetic patients, and cardiomyocyte lipid inclusions in vitro. Nutr Diabetes (2021) 11(1):8. doi: 10.1038/s41387-021-00150-7 33558456 PMC7870957

[58] Vargas-Soria M García-Alloza M Corraliza-Gómez M. Effects of diabetes on microglial physiology: a systematic review of in *vitro*, preclinical and clinical studies. J Neuroinflammation (2023) 20(1):57. doi: 10.1186/s12974-023-02740-x 36869375 PMC9983227

[59] Oshitari T. Advanced glycation end-products and diabetic neuropathy of the retina. Int J Mol Sci (2023) 24(3):2927. doi: 10.3390/ijms24032927 36769249 PMC9917392

[60] Parwani K Mandal P. Role of advanced glycation end products and insulin resistance in diabetic nephropathy. Arch Physiol Biochem (2023) 129(1):95–107. doi: 10.1080/13813455.2020.1797106 32730131

[61] O’Brien PD Hinder LM Parlee SD Hayes JM Backus C Zhang H. Dual CCR2/CCR5 antagonist treatment attenuates adipose inflammation, but not microvascular complications in ob/ob mice. Diabetes Obes Metab (2017) 19(10):1468–72. doi: 10.1111/dom.12950 PMC5610585 28332276

[62] Ma X Ma J Leng T Yuan Z Hu T Liu Q. Advances in oxidative stress in pathogenesis of diabetic kidney disease and efficacy of TCM intervention. Renal Failure. (2023) 45(1):2146512. doi: 10.1080/0886022X.2022.2146512 36762989 PMC9930779

[63] Markoulli M Flanagan J Tummanapalli SS Wu J Willcox M. The impact of diabetes on corneal nerve morphology and ocular surface integrity. Ocular Surface (2018) 16(1):45–57. doi: 10.1016/j.jtos.2017.10.006 29113918

[64] Luppi P Drain P. C-peptide antioxidant adaptive pathways in β cells and diabetes. J Internal Med (2017) 281(1):7–24. doi: 10.1111/joim.12522 27251308

[65] Wang X Li Q Han X Gong M Yu Z Xu B. Electroacupuncture alleviates diabetic peripheral neuropathy by regulating glycolipid-related GLO/AGEs/RAGE axis. Front Endocrinology (2021) 12:655591. doi: 10.3389/fendo.2021.655591 PMC8290521 34295304

[66] Papachristou S Pafili K Papanas N. Skin AGEs and diabetic neuropathy. BMC Endocrine Disord (2021) 21(1):28. doi: 10.1186/s12902-021-00697-7 PMC7903740 33622304

[67] Zaman A Arif Z Moinuddin, Akhtar K Ali WM Alam K. A study on hepatopathic, dyslipidemic and immunogenic properties of fructosylated-HSA-AGE and binding of autoantibodies in sera of obese and overweight patients with fructosylated-HSA-AGE. PloS One (2019) 14(5):e0216736. doi: 10.1371/journal.pone.0216736 31116779 PMC6530853

[68] Thornalley PJ. The potential role of thiamine (vitamin B1) in diabetic complications. Curr Diabetes Rev (2005) 1(3):287–98. doi: 10.2174/157339905774574383 18220605

[69] Brownlee M. Biochemistry and molecular cell biology of diabetic complications. Nature (2001) 414(6865):813–20. doi: 10.1038/414813a 11742414

[70] Chen YQ Su M Walia RR Hao Q Covington JW Vaughan DE. Sp1 sites mediate activation of the plasminogen activator inhibitor-1 promoter by glucose in vascular smooth muscle cells. J Biol Chem (1998) 273(14):8225–31. doi: 10.1074/jbc.273.14.8225 9525928

[71] Weigert C Brodbeck K Sawadogo M Häring HU Schleicher ED. Upstream stimulatory factor (USF) proteins induce human TGF-beta1 gene activation via the glucose-response element-1013/-1002 in mesangial cells: up-regulation of USF activity by the hexosamine biosynthetic pathway. J Biol Chem (2004) 279(16):15908–15. doi: 10.1074/jbc.M313524200 14757763

[72] Hafer-Macko CE Ivey FM Sorkin JD Macko RF. Microvascular tissue plasminogen activator is reduced in diabetic neuropathy. Neurology (2007) 69(3):268–74. doi: 10.1212/01.wnl.0000266391.20707.83 17636064

[73] Yao K Tan J Gu WZ Ye PP Wang KJ. Reactive oxygen species mediates the apoptosis induced by transforming growth factor beta(2) in human lens epithelial cells. Biochem Biophys Res Commun (2007) 354(1):278–83. doi: 10.1016/j.bbrc.2006.12.198 17217916

[74] Le Rhun Y Kirkland JB Shah GM. Cellular responses to DNA damage in the absence of Poly(ADP-ribose) polymerase. Biochem Biophys Res Commun (1998) 245(1):1–10. doi: 10.1006/bbrc.1998.8257 9535773

[75] de Murcia JM Niedergang C Trucco C Ricoul M Dutrillaux B Mark M. Requirement of poly(ADP-ribose) polymerase in recovery from DNA damage in mice and in cells. Proc Natl Acad Sci USA (1997) 94(14):7303–7. doi: 10.1073/pnas.94.14.7303 PMC23816 9207086

[76] de Murcia G Schreiber V Molinete M Saulier B Poch O Masson M. Structure and function of poly(ADP-ribose) polymerase. Mol Cell Biochem (1994) 138(1-2):15–24. doi: 10.1007/BF00928438 7898458

[77] Adki KM Kulkarni YA. Biomarkers in diabetic neuropathy. Arch Physiol Biochem (2023) 129(2):460–75. doi: 10.1080/13813455.2020.1837183 33186087

[78] Rudat V Küpper JH Weber KJ. Trans-dominant inhibition of poly(ADP-ribosyl)ation leads to decreased recovery from ionizing radiation-induced cell killing. Int J Radiat Biol (1998) 73(3):325–30. doi: 10.1080/095530098142428 9525261

[79] Ehrlich W Huser H Kröger H. Inhibition of the induction of collagenase by interleukin-1 beta in cultured rabbit synovial fibroblasts after treatment with the poly(ADP-ribose)-polymerase inhibitor 3-aminobenzamide. Rheumatol Int (1995) 15(4):171–2. doi: 10.1007/BF00301776 8835300

[80] Pacher P Szabó C. Role of poly(ADP-ribose) polymerase-1 activation in the pathogenesis of diabetic complications: endothelial dysfunction, as a common underlying theme. Antioxid Redox Signal (2005) 7(11-12):1568–80. doi: 10.1089/ars.2005.7.1568 PMC2228261 16356120

[81] Nikitin AG Chudakova DA Strokov IA Bursa TR Chistiakov DA Nosikov VV. Leu54Phe and Val762Ala polymorphisms in the poly(ADP-ribose)polymerase-1 gene are associated with diabetic polyneuropathy in Russian type 1 diabetic patients. Diabetes Res Clin Practice (2008) 79(3):446–52. doi: 10.1016/j.diabres.2007.10.020 18054108

[82] Drel VR Pacher P Stavniichuk R Xu W Zhang J Kuchmerovska TM. Poly(ADP-ribose)polymerase inhibition counteracts renal hypertrophy and multiple manifestations of peripheral neuropathy in diabetic Akita mice. Int J Mol Med (2011) 28(4):629–35. doi: 10.3892/ijmm.2011.709 PMC3375175 21617845

[83] Obrosova IG Xu W Lyzogubov VV Ilnytska O Mashtalir N Vareniuk I. PARP inhibition or gene deficiency counteracts intraepidermal nerve fiber loss and neuropathic pain in advanced diabetic neuropathy. Free Radical Biol Med (2008) 44(6):972–81. doi: 10.1016/j.freeradbiomed.2007.09.013 PMC3057075 17976390

[84] Zhang W Murakawa Y Wozniak KM Slusher B Sima AA. The preventive and therapeutic effects of GCPII (NAALADase) inhibition on painful and sensory diabetic neuropathy. J Neurological Sci (2006) 247(2):217–23. doi: 10.1016/j.jns.2006.05.052 16780883

[85] Drel VR Lupachyk S Shevalye H Vareniuk I Xu W Zhang J. New therapeutic and biomarker discovery for peripheral diabetic neuropathy: PARP inhibitor, nitrotyrosine, and tumor necrosis factor-{alpha}. Endocrinology (2010) 151(6):2547–55. doi: 10.1210/en.2009-1342 PMC2875829 20357221

[86] Nishikawa T Edelstein D Du XL Yamagishi S Matsumura T Kaneda Y. Normalizing mitochondrial superoxide production blocks three pathways of hyperglycaemic damage. Nature (2000) 404(6779):787–90. doi: 10.1038/35008121 10783895

[87] Obrosova IG Drel VR Pacher P Ilnytska O Wang ZQ Stevens MJ. Oxidative-nitrosative stress and poly(ADP-ribose) polymerase (PARP) activation in experimental diabetic neuropathy: the relation is revisited. Diabetes (2005) 54(12):3435–41. doi: 10.2337/diabetes.54.12.3435 PMC2228259 16306359

[88] Wang Y Schmeichel AM Iida H Schmelzer JD Low PA. Ischemia-reperfusion injury causes oxidative stress and apoptosis of Schwann cell in acute and chronic experimental diabetic neuropathy. Antioxid Redox Signal (2005) 7(11-12):1513–20. doi: 10.1089/ars.2005.7.1513 16356115

[89] Shakeel M. Recent advances in understanding the role of oxidative stress in diabetic neuropathy. Diabetes Metab Syndrome (2015) 9(4):373–8. doi: 10.1016/j.dsx.2014.04.029 25470637

[90] Dewanjee S Das S Das AK Bhattacharjee N Dihingia A Dua TK. Molecular mechanism of diabetic neuropathy and its pharmacotherapeutic targets. Eur J Pharmacol (2018) 833:472–523. doi: 10.1016/j.ejphar.2018.06.034 29966615

[91] Obrosova IG Li F Abatan OI Forsell MA Komjáti K Pacher P. Role of poly(ADP-ribose) polymerase activation in diabetic neuropathy. Diabetes (2004) 53(3):711–20. doi: 10.2337/diabetes.53.3.711 14988256

[92] Ilnytska O Lyzogubov VV Stevens MJ Drel VR Mashtalir N Pacher P. Poly(ADP-ribose) polymerase inhibition alleviates experimental diabetic sensory neuropathy. Diabetes (2006) 55(6):1686–94. doi: 10.2337/db06-0067 PMC2228258 16731831

[93] Negi G Kumar A Sharma SS. Concurrent targeting of nitrosative stress-PARP pathway corrects functional, behavioral and biochemical deficits in experimental diabetic neuropathy. Biochem Biophys Res Commun (2010) 391(1):102–6. doi: 10.1016/j.bbrc.2009.11.010 19900402

[94] Lupachyk S Shevalye H Maksimchyk Y Drel VR Obrosova IG. PARP inhibition alleviates diabetes-induced systemic oxidative stress and neural tissue 4-hydroxynonenal adduct accumulation: correlation with peripheral nerve function. Free Radical Biol Med (2011) 50(10):1400–9. doi: 10.1016/j.freeradbiomed.2011.01.037 PMC3081984 21300148

[95] Agrawal R Reno CM Sharma S Christensen C Huang Y Fisher SJ. Insulin action in the brain regulates both central and peripheral functions. Am J Physiol Endocrinol Metab (2021) 321(1):E156–e63. doi: 10.1152/ajpendo.00642.2020 PMC8321819 34056920

[96] Grote CW Wright DE. A role for insulin in diabetic neuropathy. Front Neurosci (2016) 10:581. doi: 10.3389/fnins.2016.00581 28066166 PMC5179551

[97] Shetter AR Muttagi G Sagar CB. Expression and localization of insulin receptors in dissociated primary cultures of rat Schwann cells. Cell Biol Int (2011) 35(3):299–304. doi: 10.1042/CBI20100523 20977434

[98] Huang TJ Verkhratsky A Fernyhough P. Insulin enhances mitochondrial inner membrane potential and increases ATP levels through phosphoinositide 3-kinase in adult sensory neurons. Mol Cell Neurosci (2005) 28(1):42–54. doi: 10.1016/j.mcn.2004.08.009 15607940

[99] Ozaki K Yamano S Matsuura T Narama I. Insulin-ameliorated peripheral motor neuropathy in spontaneously diabetic WBN/Kob rats. J Veterinary Med Science (2013) 75(10):1323–8. doi: 10.1292/jvms.13-0184 PMC3942929 23748976

[100] Sirisha A Gaur GS Pal P Bobby Z Balakumar B Pal GK. Effect of honey and insulin treatment on oxidative stress and nerve conduction in an experimental model of diabetic neuropathy Wistar rats. PloS One (2021) 16(1):e0245395. doi: 10.1371/journal.pone.0245395 33449943 PMC7810291

[101] Rachana KS Manu MS Advirao GM. Insulin-induced upregulation of lipoprotein lipase in Schwann cells during diabetic peripheral neuropathy. Diabetes Metab Syndrome. (2018) 12(4):525–30. doi: 10.1016/j.dsx.2018.03.017 29602762

[102] Shettar A Muttagi G. Developmental regulation of insulin receptor gene in sciatic nerves and role of insulin on glycoprotein P0 in the Schwann cells. Peptides (2012) 36(1):46–53. doi: 10.1016/j.peptides.2012.04.012 22564491

[103] Hackett AR Strickland A Milbrandt J. Disrupting insulin signaling in Schwann cells impairs myelination and induces a sensory neuropathy. Glia. (2020) 68(5):963–78. doi: 10.1002/glia.23755 PMC7067678 31758725

[104] Brussee V Cunningham FA Zochodne DW. Direct insulin signaling of neurons reverses diabetic neuropathy. Diabetes. (2004) 53(7):1824–30. doi: 10.2337/diabetes.53.7.1824 15220207

[105] Boucher J Kleinridders A Kahn CR. Insulin receptor signaling in normal and insulin-resistant states. Cold Spring Harbor Perspect Biol (2014) 6(1):a009191. doi: 10.1101/cshperspect.a009191 PMC3941218 24384568

[106] Korhonen JM Saïd FA Wong AJ Kaplan DR. Gab1 mediates neurite outgrowth, DNA synthesis, and survival in PC12 cells. J Biol Chem (1999) 274(52):37307–14. doi: 10.1074/jbc.274.52.37307 10601297

[107] Soltoff SP Rabin SL Cantley LC Kaplan DR. Nerve growth factor promotes the activation of phosphatidylinositol 3-kinase and its association with the trk tyrosine kinase. J Biol Chem (1992) 267(24):17472–7. doi: 10.1016/S0021-9258(18)41950-3 1380963

[108] Ogata T Iijima S Hoshikawa S Miura T Yamamoto S Oda H. Opposing extracellular signal-regulated kinase and Akt pathways control Schwann cell myelination. J Neurosci (2004) 24(30):6724–32. doi: 10.1523/JNEUROSCI.5520-03.2004 PMC6729716 15282275

[109] Aghanoori MR Smith DR Roy Chowdhury S Sabbir MG Calcutt NA Fernyhough P. Insulin prevents aberrant mitochondrial phenotype in sensory neurons of type 1 diabetic rats. Exp Neurology (2017) 297:148–57. doi: 10.1016/j.expneurol.2017.08.005 PMC5612919 28803751

[110] Grote CW Morris JK Ryals JM Geiger PC Wright DE. Insulin receptor substrate 2 expression and involvement in neuronal insulin resistance in diabetic neuropathy. Exp Diabetes Res (2011) 2011:212571. doi: 10.1155/2011/212571 21754917 PMC3132877

[111] Guo G Kan M Martinez JA Zochodne DW. Local insulin and the rapid regrowth of diabetic epidermal axons. Neurobiol Disease (2011) 43(2):414–21. doi: 10.1016/j.nbd.2011.04.012 21530660

[112] Grote CW Groover AL Ryals JM Geiger PC Feldman EL Wright DE. Peripheral nervous system insulin resistance in ob/ob mice. Acta Neuropathologica Commun (2013) 1:15. doi: 10.1186/2051-5960-1-15 PMC3893412 24252636

[113] Cameron NE Eaton SE Cotter MA Tesfaye S. Vascular factors and metabolic interactions in the pathogenesis of diabetic neuropathy. Diabetologia (2001) 44(11):1973–88. doi: 10.1007/s001250100001 11719828

[114] Tuck RR Schmelzer JD Low PA. Endoneurial blood flow and oxygen tension in the sciatic nerves of rats with experimental diabetic neuropathy. Brain (1984) 107(Pt 3):935–50. doi: 10.1093/brain/107.3.935 6478183

[115] Østergaard L Finnerup NB Terkelsen AJ Olesen RA Drasbek KR Knudsen L. The effects of capillary dysfunction on oxygen and glucose extraction in diabetic neuropathy. Diabetologia. (2015) 58(4):666–77. doi: 10.1007/s00125-014-3461-z PMC4351434 25512003

[116] Takeshita Y Sato R Kanda T. Blood-nerve barrier (BNB) pathology in diabetic peripheral neuropathy and *in vitro* human BNB model. Int J Mol Sci (2020) 22(1):62. doi: 10.3390/ijms22010062 33374622 PMC7793499

[117] Giannini C Dyck PJ. Basement membrane reduplication and pericyte degeneration precede development of diabetic polyneuropathy and are associated with its severity. Ann Neurology (1995) 37(4):498–504. doi: 10.1002/ana.410370412 7717686

[118] Gonçalves NP Vægter CB Andersen H Østergaard L Calcutt NA Jensen TS. Schwann cell interactions with axons and microvessels in diabetic neuropathy. Nat Reviews: Neurology (2017) 13(3):135–47. doi: 10.1038/nrneurol.2016.201 PMC7391875 28134254

[119] Jende JME Mooshage C Kender Z Schimpfle L Juerchott A Heiland S. Sciatic nerve microvascular permeability in type 2 diabetes decreased in patients with neuropathy. Ann Clin Trans Neurology (2022) 9(6):830–40. doi: 10.1002/acn3.51563 PMC9186151 35488789

[120] Zochodne DW. The challenges of diabetic polyneuropathy: a brief update. Curr Opin Neurology (2019) 32(5):666–75. doi: 10.1097/WCO.0000000000000723 31306212

[121] Stirban A. Microvascular dysfunction in the context of diabetic neuropathy. Curr Diabetes Rep (2014) 14(11):541. doi: 10.1007/s11892-014-0541-x 25189434

[122] Reynès C Beaume JB Latil-Plat F Ennaifer H Rocher L Antoine-Jonville S. Concomitant peripheral neuropathy and type 2 diabetes impairs postexercise cutaneous perfusion and flowmotion. J Clin Endocrinol Metab (2021) 106(10):e3979–e89. doi: 10.1210/clinem/dgab414 34111245

[123] Zhang H Nie X Shi X Zhao J Chen Y Yao Q. Regulatory mechanisms of the wnt/β-catenin pathway in diabetic cutaneous ulcers. Front Pharmacol (2018) 9:1114. doi: 10.3389/fphar.2018.01114 30386236 PMC6199358

[124] Napolitano T Silvano S Ayachi C Plaisant M Sousa-Da-Veiga A Fofo H. Wnt pathway in pancreatic development and pathophysiology. Cells (2023) 12(4):565. doi: 10.3390/cells12040565 36831232 PMC9954665

[125] Marini F Giusti F Palmini G Brandi ML. Role of Wnt signaling and sclerostin in bone and as therapeutic targets in skeletal disorders. Osteoporosis Int (2023) 34(2):213–38. doi: 10.1007/s00198-022-06523-7 35982318

[126] van der Wal T van Amerongen R. Visualizing WNT signaling in mammalian systems. Curr Topics Dev Biol (2023) 153:61–93. doi: 10.1016/bs.ctdb.2023.02.001 36967202

[127] Du W Menjivar RE Donahue KL Kadiyala P Velez-Delgado A Brown KL. WNT signaling in the tumor microenvironment promotes immunosuppression in murine pancreatic cancer. J Exp Med (2023) 220(1):e20220503. doi: 10.1084/jem.20220503 36239683 PMC9577101

[128] Resham K Khare P Bishnoi M Sharma SS. Neuroprotective effects of isoquercitrin in diabetic neuropathy via Wnt/β-catenin signaling pathway inhibition. Biofactors (2020) 46(3):411–20. doi: 10.1002/biof.1615 31960520

[129] Pan S Hada SS Liu Y Hu C Zhou M Zheng S. Human placenta-derived mesenchymal stem cells ameliorate diabetic neuropathy via wnt signaling pathway. Stem Cells Int (2022) 2022:6897056. doi: 10.1155/2022/6897056 36440182 PMC9683984

[130] Yu J Zhao Y Xu L Li W Zhang H Ping F. Liraglutide attenuates hepatic oxidative stress, inflammation, and apoptosis in streptozotocin-induced diabetic mice by modulating the wnt/β-catenin signaling pathway. Mediators Inflammation (2023) 2023:8974960. doi: 10.1155/2023/8974960 PMC9899592 36756089

[131] Hutton SR Otis JM Kim EM Lamsal Y Stuber GD Snider WD. ERK/MAPK signaling is required for pathway-specific striatal motor functions. J Neurosci (2017) 37(34):8102–15. doi: 10.1523/JNEUROSCI.0473-17.2017 PMC5566864 28733355

[132] Vieira WF Malange KF de Magalhães SF Lemes JBP Dos Santos GG Nishijima CM. Anti-hyperalgesic effects of photobiomodulation therapy (904 nm) on streptozotocin-induced diabetic neuropathy imply MAPK pathway and calcium dynamics modulation. Sci Rep (2022) 12(1):16730. doi: 10.1038/s41598-022-19947-2 36202956 PMC9537322

[133] Sanaye MM Kavishwar SA. Diabetic neuropathy: review on molecular mechanisms. Curr Mol Med (2023) 23(2):97–110. doi: 10.2174/1566524021666210816093111 34397329

[134] Suo J Wang M Zhang P Lu Y Xu R Zhang L. Siwei Jianbu decoction improves painful paclitaxel-induced peripheral neuropathy in mouse model by modulating the NF-κB and MAPK signaling pathways. Regenerative Med Res (2020) 8:2. doi: 10.1051/rmr/200001 PMC7583579 33095154

[135] Bengal E Aviram S Hayek T. p38 MAPK in glucose metabolism of skeletal muscle: beneficial or harmful? Int J Mol Sci (2020) 21(18):6480. doi: 10.3390/ijms21186480 32899870 PMC7555282

[136] Li R Li Y Wu Y Zhao Y Chen H Yuan Y. Heparin-poloxamer thermosensitive hydrogel loaded with bFGF and NGF enhances peripheral nerve regeneration in diabetic rats. Biomaterials (2018) 168:24–37. doi: 10.1016/j.biomaterials.2018.03.044 29609091 PMC5935004

[137] Xu J Ji J Yan XH. Cross-talk between AMPK and mTOR in regulating energy balance. Crit Rev Food Sci Nutr (2012) 52(5):373–81. doi: 10.1080/10408398.2010.500245 22369257

[138] Towns R Kabeya Y Yoshimori T Guo C Shangguan Y Hong S. Sera from patients with type 2 diabetes and neuropathy induce autophagy and colocalization with mitochondria in SY5Y cells. Autophagy (2005) 1(3):163–70. doi: 10.4161/auto.1.3.2068 16874076

[139] Tatsumi Y Kato A Niimi N Yako H Himeno T Kondo M. Docosahexaenoic acid suppresses oxidative stress-induced autophagy and cell death via the AMPK-dependent signaling pathway in immortalized fischer rat schwann cells 1. Int J Mol Sci (2022) 23(8):4405. doi: 10.3390/ijms23084405 35457223 PMC9027959

[140] Liu SY Chen L Li XC Hu QK He LJ. *Lycium barbarum* polysaccharide protects diabetic peripheral neuropathy by enhancing autophagy via mTOR/p70S6K inhibition in Streptozotocin-induced diabetic rats. J Chem Neuroanatomy (2018) 89:37–42. doi: 10.1016/j.jchemneu.2017.12.011 29294366

[141] Yin Y Qu H Yang Q Fang Z Gao R. Astragaloside IV alleviates Schwann cell injury in diabetic peripheral neuropathy by regulating microRNA-155-mediated autophagy. Phytomedicine. (2021) 92:153749. doi: 10.1016/j.phymed.2021.153749 34601220

[142] Dong J Li H Bai Y Wu C. Muscone ameliorates diabetic peripheral neuropathy through activating AKT/mTOR signalling pathway. J Pharm Pharmacol (2019) 71(11):1706–13. doi: 10.1111/jphp.13157 31468549

[143] Beirowski B Wong KM Babetto E Milbrandt J. mTORC1 promotes proliferation of immature Schwann cells and myelin growth of differentiated Schwann cells. Proc Natl Acad Sci USA (2017) 114(21):E4261–e70. doi: 10.1073/pnas.1620761114 PMC5448230 28484008

[144] Zhu L Hao J Cheng M Zhang C Huo C Liu Y. Hyperglycemia-induced Bcl-2/Bax-mediated apoptosis of Schwann cells via mTORC1/S6K1 inhibition in diabetic peripheral neuropathy. Exp Cell Res (2018) 367(2):186–95. doi: 10.1016/j.yexcr.2018.03.034 29621478

[145] Cheng M Lv X Zhang C Du W Liu Y Zhu L. DNMT1, a novel regulator mediating mTORC1/mTORC2 pathway-induced NGF expression in schwann cells. Neurochemical Res (2018) 43(11):2141–54. doi: 10.1007/s11064-018-2637-1 30229399

[146] Maiese K. Novel nervous and multi-system regenerative therapeutic strategies for diabetes mellitus with mTOR. Neural Regeneration Res (2016) 11(3):372–85. doi: 10.4103/1673-5374.179032 PMC4828986 27127460

[147] Zhang CH Lv X Du W Cheng MJ Liu YP Zhu L. The Akt/mTOR cascade mediates high glucose-induced reductions in BDNF via DNMT1 in Schwann cells in diabetic peripheral neuropathy. Exp Cell Res (2019) 383(1):111502. doi: 10.1016/j.yexcr.2019.111502 31323191

[148] Beirowski B. The LKB1-AMPK and mTORC1 Metabolic Signaling Networks in Schwann Cells Control Axon Integrity and Myelination: Assembling and upholding nerves by metabolic signaling in Schwann cells. Bioessays (2019) 41(1):e1800075. doi: 10.1002/bies.201800075 30537168

[149] Allam MA Nassar YA Shabana HS Mostafa S Khalil F Zidan H. Prevalence and clinical significance of subclinical hypothyroidism in diabetic peripheral neuropathy. Int J Gen Med (2021) 14:7755–61. doi: 10.2147/IJGM.S337779 PMC8579825 34785933

[150] Zhao W Zeng H Zhang X Liu F Pan J Zhao J. A high thyroid stimulating hormone level is associated with diabetic peripheral neuropathy in type 2 diabetes patients. Diabetes Res Clin Practice (2016) 115:122–9. doi: 10.1016/j.diabres.2016.01.018 26822260

[151] Hu Y Hu Z Tang W Liu W Wu X Pan C. Association of thyroid hormone levels with microvascular complications in euthyroid type 2 diabetes mellitus patients. Diabetes Metab Syndrome Obes (2022) 15:2467–77. doi: 10.2147/DMSO.S354872 PMC9380826 35982763

[152] Pramanik S Ghosh S Mukhopadhyay P Bhattacharjee R Mukherjee B Mondal SA. Thyroid status in patients with type 2 diabetes attending a tertiary care hospital in eastern India. Indian J Endocrinol Metab (2018) 22(1):112–5. doi: 10.4103/ijem.IJEM_572_17 PMC5838889 29535948

[153] Salman Jasim H Khalid Shafeeq N Abass EAA. Vitamin D level and its relation with the newly diagnosed diabetic neuropathy in women with hypothyroidism. Arch Razi Institute (2022) 77(3):1139–45. doi: 10.22092/ARI.2022.357389.2029 PMC9759228 36618309

[154] Gul H Odabasi Z Yildiz O Ozata M Deniz G Vural O. Beneficial effect of thyrotropin-releasing hormone on neuropathy in diabetic rats. Diabetes Res Clin Practice (1999) 44(2):93–100. doi: 10.1016/S0168-8227(99)00028-5 10414927

[155] Fan J Pan Q Gao Q Li W Xiao F Guo L. TSH combined with TSHR aggravates diabetic peripheral neuropathy by promoting oxidative stress and apoptosis in schwann cells. Oxid Med Cell Longevity (2021) 2021:2482453. doi: 10.1155/2021/2482453 PMC8601831 34804362

[156] Ziegler D Papanas N Schnell O Nguyen BDT Nguyen KT Kulkantrakorn K. Current concepts in the management of diabetic polyneuropathy. J Diabetes Invest (2021) 12(4):464–75. doi: 10.1111/jdi.13401 PMC8015839 32918837

[157] Feldman EL Stevens MJ Thomas PK Brown MB Canal N Greene DA. A practical two-step quantitative clinical and electrophysiological assessment for the diagnosis and staging of diabetic neuropathy. Diabetes Care (1994) 17(11):1281–9. doi: 10.2337/diacare.17.11.1281 7821168

[158] Young MJ Boulton AJ MacLeod AF Williams DR Sonksen PH. A multicentre study of the prevalence of diabetic peripheral neuropathy in the United Kingdom hospital clinic population. Diabetologia (1993) 36(2):150–4. doi: 10.1007/BF00400697 8458529

[159] Ziegler D Hanefeld M Ruhnau KJ Meissner HP Lobisch M Schutte K. Treatment of symptomatic diabetic peripheral neuropathy with the anti-oxidant alpha-lipoic acid. A 3-week multicentre randomized controlled trial (ALADIN Study). Diabetologia (1995) 38(12):1425–33. doi: 10.1007/BF00400603 8786016

[160] Wang D Wang C Duan X Yang Z Bai Z Hu H. MR T2 value of the tibial nerve can be used as a potential non-invasive and quantitative biomarker for the diagnosis of diabetic peripheral neuropathy. Eur Radiology (2018) 28(3):1234–41. doi: 10.1007/s00330-017-5043-1 29038932

[161] Kazamel M Stino AM Smith AG. Metabolic syndrome and peripheral neuropathy. Muscle Nerve. (2021) 63(3):285–93. doi: 10.1002/mus.27086 33098165

[162] Stratton IM Adler AI Neil HA Matthews DR Manley SE Cull CA. Association of glycaemia with macrovascular and microvascular complications of type 2 diabetes (UKPDS 35): prospective observational study. Br Med J (2000) 321(7258):405–12. doi: 10.1136/bmj.321.7258.405 PMC27454 10938048

[163] Hanewinckel R Drenthen J Ligthart S Dehghan A Franco OH Hofman A. Metabolic syndrome is related to polyneuropathy and impaired peripheral nerve function: a prospective population-based cohort study. J Neurology Neurosurg Psychiatry (2016) 87(12):1336–42. doi: 10.1136/jnnp-2016-314171 27656045

[164] Callaghan BC Xia R Reynolds E Banerjee M Rothberg AE Burant CF. Association between metabolic syndrome components and polyneuropathy in an obese population. JAMA Neurol (2016) 73(12):1468–76. doi: 10.1001/jamaneurol.2016.3745 PMC5217829 27802497

[165] Callaghan BC Xia R Banerjee M de Rekeneire N Harris TB Newman AB. Metabolic syndrome components are associated with symptomatic polyneuropathy independent of glycemic status. Diabetes Care (2016) 39(5):801–7. doi: 10.2337/dc16-0081 PMC4839175 26965720

[166] Harati Y Gooch C Swenson M Edelman S Greene D Raskin P. Double-blind randomized trial of tramadol for the treatment of the pain of diabetic neuropathy. Neurology. (1998) 50(6):1842–6. doi: 10.1212/WNL.50.6.1842 9633738

[167] Vinik AI Perrot S Vinik EJ Pazdera L Jacobs H Stoker M. Capsaicin 8% patch repeat treatment plus standard of care (SOC) versus SOC alone in painful diabetic peripheral neuropathy: a randomised, 52-week, open-label, safety study. BMC Neurol (2016) 16(1):251. doi: 10.1186/s12883-016-0752-7 27919222 PMC5139122

[168] Rehm S Binder A Baron R. Post-herpetic neuralgia: 5% lidocaine medicated plaster, pregabalin, or a combination of both? A randomized, open, clinical effectiveness study. Curr Med Res Opinion (2010) 26(7):1607–19. doi: 10.1185/03007995.2010.483675 20429825

[169] Zhang Y Fan D Zhang Y Zhang S Wang H Liu Z. Using corneal confocal microscopy to compare Mecobalamin intramuscular injections vs oral tablets in treating diabetic peripheral neuropathy: a RCT. Sci Rep (2021) 11(1):14697. doi: 10.1038/s41598-021-94284-4 34282267 PMC8290034

[170] Irving G Tanenberg RJ Raskin J Risser RC Malcolm S. Comparative safety and tolerability of duloxetine vs. pregabalin vs. duloxetine plus gabapentin in patients with diabetic peripheral neuropathic pain. Int J Clin Practice (2014) 68(9):1130–40. doi: 10.1111/ijcp.12452 24837444

[171] Mahmoudi-Nezhad M Vajdi M Farhangi MA. An updated systematic review and dose-response meta-analysis of the effects of alpha-lipoic acid supplementation on glycemic markers in adults. Nutrition (2021) 82:111041. doi: 10.1016/j.nut.2020.111041 33199187

[172] Sawangjit R Thongphui S Chaichompu W Phumart P. Efficacy and safety of mecobalamin on peripheral neuropathy: A systematic review and meta-analysis of randomized controlled trials. J Altern Complementary Med (2020) 26(12):1117–29. doi: 10.1089/acm.2020.0068 32716261

[173] Xu Q Pan J Yu J Liu X Liu L Zuo X. Meta-analysis of methylcobalamin alone and in combination with lipoic acid in patients with diabetic peripheral neuropathy. Diabetes Res Clin Practice (2013) 101(2):99–105. doi: 10.1016/j.diabres.2013.03.033 23664235

[174] Mimenza Alvarado A Aguilar Navarro S. Clinical trial assessing the efficacy of gabapentin plus B complex (B1/B12) versus pregabalin for treating painful diabetic neuropathy. J Diabetes Res (2016) 2016:4078695. doi: 10.1155/2016/4078695 26885528 PMC4739211

[175] Jiang DQ Li MX Ma YJ Wang Y Wang Y. Efficacy and safety of prostaglandin E1 plus lipoic acid combination therapy versus monotherapy for patients with diabetic peripheral neuropathy. J Clin Neurosci (2016) 27:8–16. doi: 10.1016/j.jocn.2015.07.028 26775115

[176] Jiang DQ Zhao SH Li MX Jiang LL Wang Y Wang Y. Prostaglandin E1 plus methylcobalamin combination therapy versus prostaglandin E1 monotherapy for patients with diabetic peripheral neuropathy: A meta-analysis of randomized controlled trials. Med (Baltimore) (2018) 97(44):e13020. doi: 10.1097/MD.0000000000013020 PMC6221723 30383660

[177] Amato Nesbit S Sharma R Waldfogel JM Zhang A Bennett WL Yeh HC. Non-pharmacologic treatments for symptoms of diabetic peripheral neuropathy: a systematic review. Curr Med Res Opinion (2019) 35(1):15–25. doi: 10.1080/03007995.2018.1497958 30114983

[178] Petersen EA Stauss TG Scowcroft JA Brooks ES White JL Sills SM. Effect of high-frequency (10-kHz) spinal cord stimulation in patients with painful diabetic neuropathy. JAMA Neurol (2021) 78(6):687–98. doi: 10.26226/morressier.617c37317c09fc044a9751b7 PMC8022268 33818600

